# Human metapneumovirus respiratory infection affects both innate and adaptive intestinal immunity

**DOI:** 10.3389/fimmu.2024.1330209

**Published:** 2024-02-02

**Authors:** Javiera Sepúlveda-Alfaro, Eduardo A. Catalán, Omar P. Vallejos, Ignacio Ramos-Tapia, Cristóbal Madrid-Muñoz, María J. Mendoza-León, Isidora D. Suazo, Elizabeth Rivera-Asin, Pedro H. Silva, Oscar Alvarez-Mardones, Daniela P. Castillo-Godoy, Claudia A. Riedel, Katina Schinnerling, Juan A. Ugalde, Jorge A. Soto, Susan M. Bueno, Alexis M. Kalergis, Felipe Melo-Gonzalez

**Affiliations:** ^1^ Millennium Institute on Immunology and Immunotherapy, Departamento de Genética Molecular y Microbiología, Facultad de Ciencias Biológicas, Pontificia Universidad Católica de Chile, Santiago, Chile; ^2^ Millennium Institute on Immunology and Immunotherapy, Departamento de Ciencias Biológicas, Facultad de Ciencias de la Vida, Universidad Andrés Bello, Santiago, Chile; ^3^ Center for Bioinformatics and Integrative Biology, Facultad de Ciencias de la Vida, Universidad Andrés Bello, Santiago, Chile; ^4^ Facultad de Ciencias de la Vida, Universidad Andrés Bello, Santiago, Chile; ^5^ Departamento de Endocrinología, Facultad de Medicina, Pontificia Universidad Católica de Chile, Santiago, Chile

**Keywords:** HMPV, lung-gut axis, monocytes, CD8 + T cells, microbiota

## Abstract

**Introduction:**

Respiratory infections are one of the leading causes of morbidity and mortality worldwide, mainly in children, immunocompromised people, and the elderly. Several respiratory viruses can induce intestinal inflammation and alterations in intestinal microbiota composition. Human metapneumovirus (HMPV) is one of the major respiratory viruses contributing to infant mortality in children under 5 years of age worldwide, and the effect of this infection at the gut level has not been studied.

**Methods:**

Here, we evaluated the distal effects of HMPV infection on intestinal microbiota and inflammation in a murine model, analyzing several post-infection times (days 1, 3, and 5). Six to eight-week-old C57BL/6 mice were infected intranasally with HMPV, and mice inoculated with a non-infectious supernatant (Mock) were used as a control group.

**Results:**

We did not detect HMPV viral load in the intestine, but we observed significant changes in the transcription of IFN-γ in the colon, analyzed by qPCR, at day 1 post-infection as compared to the control group. Furthermore, we analyzed the frequencies of different innate and adaptive immune cells in the colonic lamina propria, using flow cytometry. The frequency of monocyte populations was altered in the colon of HMPV -infected mice at days 1 and 3, with no significant difference from control mice at day 5 post-infection. Moreover, colonic CD8^+^ T cells and memory precursor effector CD8^+^ T cells were significantly increased in HMPV-infected mice at day 5, suggesting that HMPV may also alter intestinal adaptive immunity. Additionally, we did not find alterations in antimicrobial peptide expression, the frequency of colonic IgA^+^ plasma cells, and levels of fecal IgA. Some minor alterations in the fecal microbiota composition of HMPV -infected mice were detected using 16s rRNA sequencing. However, no significant differences were found in β-diversity and relative abundance at the genus level.

**Discussion:**

To our knowledge, this is the first report describing the alterations in intestinal immunity following respiratory infection with HMPV infection. These effects do not seem to be mediated by direct viral infection in the intestinal tract. Our results indicate that HMPV can affect colonic innate and adaptive immunity but does not significantly alter the microbiota composition, and further research is required to understand the mechanisms inducing these distal effects in the intestine.

## Introduction

Respiratory diseases are a major health burden worldwide, causing morbidity and mortality in young infants and the elderly. Pneumonia is a lower respiratory tract disease caused by multiple pathogens, including bacteria and viruses (1). Increasing evidence indicates that respiratory viral infections such as influenza and human respiratory syncytial virus (HRSV) can also cause intestinal immune responses, inflammation, and changes in the gut microbiota, suggesting that viral infections in the lung may lead to systemic comorbidities (2–6). Evidence from influenza infection suggests that these distal effects in the intestine are mediated by systemic inflammatory signals or the migration of lung-derived immune cells such as T cells rather than direct viral infection in the gut (2). On the other hand, HRSV respiratory infection effects in the intestinal tract are attributed to altered food intake during viral infection (6). Therefore, it is important to understand whether other respiratory viruses may impact intestinal homeostasis.

One of the most prevalent and poorly understood viruses that cause community-acquired pneumonia is human metapneumovirus (HMPV). HMPV was discovered in 2001 and has become the second cause of pneumonia and bronchiolitis, particularly in children under 5 years old (7, 8). HMPV induces a rapid immune response with a robust innate component, accompanied by the production of inflammatory cytokines, particularly of a type 2 profile (9). TSLP, IL-25, and IL-33 are known inducers of type 2 cytokine responses by innate immune cells, triggering hypersecretion of mucus and lung allergic inflammatory responses (9, 10). In addition, pro-inflammatory cytokines activate the production of chemokines that recruit neutrophils into the lung (9, 10). Still, although these cells help to control infection and clear the pathogen, they also contribute to lung inflammation and tissue damage. Indeed, neutrophil depletion seems protective, as mice exhibit decreased inflammation and reduced viral load in murine models (11). Other innate and adaptive immune cells, such as alveolar macrophages, dendritic cells, NK cells, and cytotoxic T cells, exhibit significant antiviral responses against HMPV (12–15). Inducting inappropriate or exacerbated immune responses against HMPV may lead to chronic diseases, including chronic obstructive pulmonary disease (COPD) and increased susceptibility to asthma (16, 17). Diarrhea has been reported as a symptom in patients with HMPV, suggesting that HMPV infection can induce intestinal inflammation, but it has not been proven in animal models (16, 18, 19).

Based on this evidence, we hypothesized that respiratory infection with HMPV distally affects intestinal immunity and induces changes in the inflammatory profile and microbiota of C57BL/6 mice. Therefore, we evaluated the infectivity of HMPV in the intestine of C57BL/6 mice and corroborated infection in the lung using qPCR. Next, we evaluated the inflammatory response in the intestine in HMPV-infected mice, assessing the expression of pro-inflammatory cytokines and antimicrobial peptides using qPCR and the frequency of innate inflammatory myeloid cells and adaptive immunes cells (CD8^+^ T cells and IgA^+^ plasma cells) by flow cytometry. Finally, we evaluated changes in the fecal microbiota composition of HMPV-infected mice.

## Materials and methods

### Virus expansion and titration

HMPV serogroup A, strain CZ0107 (clinical isolate obtained from the Laboratorio de Infectología y Virología of the Hospital Clínico, Pontificia Universidad Católica de Chile) (20) was expanded using LLC-MK2 cells (American Type Culture Collection, CCL-7TM) in medium 199 (Gibco) supplemented with 1% horse serum, which were grown in T-75 bottles until reaching 80-90% confluency. To infect the cells, an MOI equal to 0.1 of HMPV in 5 mL of infection medium (medium 199 supplemented with 100 µg/ml CaCl_2_ and Trypsin 5 µg/mL) was used, and cells were incubated at 37°C for 2 h and 5% CO_2_. After 72 h, the cells were scraped and pooled to be centrifuged at 300xg for 10 min and then at 500xg for 10 min to remove cell debris. Supernatants of non-infected LLC-MK2 cells were used as a non-infectious control (Mock) and obtained as previously reported (21, 22). Virus and mock were aliquoted into cryotubes and stored at -80°C. Viral titers of supernatants were determined by immunocytochemistry in 96-well plates with LLC-MK2 cells monolayers infected with serial dilutions of the virus (21–23).

### HMPV infection

Six to eight-week-old C57BL/6 male mice were intraperitoneally anesthetized with a dose of Ketamine/Xylazine (80 mg/kg and 4 mg/kg, respectively) and then intranasally administered with 10^6^ PFU of HMPV or mock (non-infectious supernatant) in a final volume of 80 μL per mouse. Experimental parameters were assessed at days 1, 3, and 5 post-infection. Subsequently, daily monitoring and clinical score follow-up were performed until the day of euthanasia, as previously published (21). For euthanasia, a dose of Ketamine/Xylazine (80 mg/kg and 10 mg/kg, respectively) was intraperitoneally administered to the mice, followed by cervical dislocation. All mouse experiments were conducted in agreement with ethical standards and according to the local animal protection law number 20.800. Furthermore, all experimental protocols were designed according to the Sanitary Code of Terrestrial Animals of the World Organization for Animal Health (OIE, 24^a^ Edition, 2015) and were reviewed and approved by the Scientific Ethical Committee for Animal and Environment Care of the Pontificia Universidad Católica de Chile (Protocol number 181012020) and the Bioethics Committee of Universidad Andrés Bello.

### Organ and fecal collection

Samples of lung and intestine were collected for flow cytometry analysis and RNA extraction to assess viral load by qPCR and proinflammatory cytokines present in the intestine by RT-qPCR. Feces were directly collected from the colon to extract DNA for evaluating bacterial groups by qPCR and 16S sequencing and for total IgA detection.

### RNA extraction

Approximately 3 mm of tissue from the initial, middle, and final parts of the small intestine and colon were collected and stored TRIzol reagent (Invitrogen) and held at -80°C for RNA extraction. RNA extraction from the intestine and lung was performed using TRIzol reagent following the manufacturer’s protocol. RNA samples were resuspended in 100 μL of nuclease-free water, and the concentration and purity were measured using a NanoDrop 2000 Spectrophotometer (Thermo Fisher Scientific). The samples were stored at -80°C until further use.

### Quantitative PCR with reverse transcription

For the extracted intestinal RNA, real-time quantitative PCR (RT-qPCR) was performed to detect the transcription of genes encoding for *Ifng*, *Il6*, *Il23*a, *Il17a*, *RegIIIg*, and *Lcn2* using the Taqman RNA-to-Ct 1-Step kit (Applied Biosystems), following the manufacturer’s instructions for a 10 μL reaction. The transcription of the analyzed genes was normalized to the transcription of β-2-microglobulin (β2m). The Taqman probes for *Ifng* (Mm01168134_m1), *Il6* (Mm00446190_m1), *Il23a* (Mm00518984_m1), *Il17a* (Mm00439618_m1), *RegIIIg* (Mm00441127_m1), *Lcn2* (Mm00441127_m1) and *β2m* (Mm00434228_m1) were obtained from Thermo Fisher Scientific.

To detect viral titers in intestinal and lung RNA, RT-qPCR was performed to detect the transcription of genes encoding the N protein of the HMPV virus and normalized to the transcription of the β-actin gene. In this case, cDNA was prepared using the iScript™ Reverse Transcription Supermix for RT-qPCR kit (BioRad), following the manufacturer’s instructions for a 20 μL reaction. Subsequently, qPCR was performed using to detect β-actin (Forward: 5’ TCCTATGTGGGTGACGAG 3’; Reverse: 5’ CTCATTGTAGAAGGTGTGGTG 3’), HMPV-N (Forward: 5’ ACAGCAGATTCTAAGAAACTCAGG 3’; Reverse: 5’ TCTTTGTCTATCTCTTCCACCC 3’). Mucin transcription in intestinal RNA was detected using primers for Muc2 (Forward: 5’ AGAACGATGCCTACACCAAG 3’; Reverse: 5’ CATTGAAGTCCCCGCAGAG 3’) and Muc5ac (Forward: 5’ ATGGGCTGTGTTCCTGTGTC 3’; Reverse: 5’ CAGAACATGTGTTGGTGCAGTC 3’) and normalized to the transcription of the β-actin gene. Fluorescence detection was performed using the SsoAdvanced™ Universal SYBR Green Supermix kit (BioRad), following the manufacturer’s instructions for a 10 μl reaction.

### Colon lamina propria digestion

Colonic lamina propria lymphocyte preparations were performed as previously described (24). Intestinal tissue was isolated, associated fat was removed, and tissue was cut open longitudinally. Fecal samples were collected for bacterial DNA extraction, and the remaining luminal contents were removed by shaking in cold PBS. To remove epithelial cells and intraepithelial lymphocytes, the colon was incubated with stripping buffer (1X PBS containing 1 mM EDTA, 1 mM dithiothreitol, and 5% FCS) for 10 min at 37°C and then repeated for a second round of 20 min. Lamina propria cells were isolated by digesting the remaining tissue in complete RPMI (supplemented with 1% L-glutamine, 1% penicillin/streptomycin, and 10% FBS at pH 7.2) containing 2 mg/ml Collagenase D (Roche) and 20 μg/ml DNase I (Sigma-Aldrich) for 45 min at 37°C. Following the tissue digestion, liberated cells were extracted by passing the tissue and supernatant over a 70-μm nylon filter and centrifugation to isolate lamina propria lymphocytes. The cells were stored at 4°C until they were stained for flow cytometry analysis.

### Lung digestion

The lungs were cut and transferred to a 15 mL falcon tube with 5 mL of Collagenase IV (T Thermo Fisher Scientific) and incubated on a shaker for 1 h at 37°C. Then, 5 mL of 5 mM EDTA was added, agitated to stop digestion, and incubated at room temperature for 2 min. Subsequently, the supernatant was discarded, and the lung tissue was collected using a 70 μm cell strainer. The tissue was homogenized and transferred to a clean 1.5 mL tube. The tube was centrifuged at 300xg for 7 min, the supernatant was discarded, and the tissue resuspended in 1 mL of 1X ACK buffer. The suspension was incubated at room temperature for 5 min and then centrifuged at 300xg for another 7 min. The supernatant was again discarded, and the tissue was resuspended in 1 mL of 1X PBS. The cells were stored at 4°C until stained for analysis by flow cytometry.

### Flow cytometry

Cells were stained in 1X PBS containing 2% FBS and 2 mM EDTA for 30 min at 4°C and then fixed with 2% PFA for 15 min at 4°C. Single-cell preparations were stained with antibodies to the following markers: anti-Ly6C (clone HK1.4, BioLegend), anti-IgA (clone mA-6E1, eBioscience), anti-Ly6G (clone 1A8, BD), anti-SiglecF (clone E50-2440, BD), anti-CD11c (clone HL3, BD), anti-CD45 (clone 30-F11, BioLegend), anti-IA/IE (clone M5/114.15.2, BD), anti-B220 (clone RA3-6B2, BioLegend), anti-CD138 (clone 281-2, BioLegend), anti-CD64 (clone X54-5/7.1, BD), anti-CD11b (clone M1-70, BD) and anti-CD5 (clone 53-7.3, BD). Dead cells were excluded from analysis using the viability dye BD Horizon Fixable Viability Stain 510 (BD). Data were acquired in an LSRFortessa X20 cytometer (BD Biosciences) and analyzed using FlowJo v10.0.7 software (BD Biosciences).

### Fecal sample processing

Fecal samples were collected in empty and pre-weighed 1.5 mL tubes. The samples were diluted with 1X PBS to a concentration of 100 μg/μL and homogenized using a sterile plastic pestle. The tubes were then centrifuged at 50xg for 15 min, and the supernatant was carefully removed and transferred to a clean 1.5 mL tube. The supernatant was then centrifuged at 8,000xg for 5 min, and the resulting supernatant was collected in a clean 1.5 mL tube and stored at -30°C. The bacterial pellet was stored at -80°C until further use.

### Lipocalin-2 and IgA ELISA

The amount of mouse fecal Lipocalin-2 and IgA was measured using a Mouse Lipocalin-2/NGAL DuoSet ELISA (R&D Systems) and IgA Mouse Uncoated ELISA Kit (Invitrogen), respectively, according to the manufacturer’s instructions. Briefly, the plates were coated overnight with the pre-titrated purified capture antibody, and after blocking, fold serial dilutions of a standard Lipocalin-2 or IgA and fecal supernatants (diluted at 1:10 for lipocalin-2 and 1:1,000 for IgA) were incubated for 2 h at room temperature (RT) on a microplate shaker at 400 rpm. For Lipocalin-2, after washing the plate, a biotinylated rat anti-mouse Lipocalin-2 was incubated at RT for 2 h with shaking at 400 rpm followed by incubation with streptavidin-HRP for 20 min. For IgA detection, pre-titrated, HRP-conjugated anti-mouse IgA polyclonal antibody was incubated at RT for 1 h with shaking at 400 rpm. After washing the plate, the Tetramethylbenzidine (TMB) substrate solution was added and incubated at RT for 15 min, and the reaction was stopped using H_2_SO_4_ 1N. The optical densities were measured at 450 nm with a correction of 570 nm using a microplate spectrophotometer (Agilent BioTek Epoch).

### Bacterial DNA extraction

Bacterial DNA extraction from the fecal samples was performed using the DNeasy PowerSoil Pro Kit (Qiagen) following the manufacturer’s instructions. The concentration and purity of the extracted DNA were measured using a NanoDrop 2000 Spectrophotometer (Thermo Fisher Scientific), and the DNA was stored at -80°C until further use.

### 16S sequencing

To identify the intestinal microbiome of mock-treated and HMPV-infected mice, bacterial DNA extracted from fecal samples was submitted to 16S amplicon sequencing using the Illumina massively parallel sequencing technique at Zymo Research (Irvine, CA). Fecal DNA was amplified using 16S primers designed by Zymo Research, targeting the hypervariable V3-V4 regions of the 16S rRNA gene. The sequencing library was prepared in real-time PCR machines, quantified using fluorescence readings, and processed with the Select-a-Size DNA Clean & Concentrator kit™ (Zymo Research). Quality control was performed in a TapeStation^®^ system (Agilent Technologies) and by fluorometric quantification using a Qubit^®^ fluorometer (Thermo Fisher Scientific). The resulting library was sequenced on Illumina^®^ MiSeq™ with a v3 reagent kit (600 cycles) (Illumina Incorporated).

### Microbiota analysis

16S rRNA amplicon sequence variants (ASV) in each sample were inferred using DADA2 version 1.28 (25). Forward reads are maintained to 280 pb, reverse reads were trimmed at 220 bp to maintain quality over PHRED 25 and filtered using the following parameters: maxN=0, maxEE=c (2, 3), truncQ=2, trimLeft=c (20, 21), rm.phix=TRUE. Error rate learning, dereplication, and read merging were performed using the default software settings. Taxonomy was assigned using the Silva database (version 138.1) (26). Multiple sequence alignment was carried out using the AlignSeqs function of the Decipher package (27), and the phylogenetic tree was inferred using the R PHANGORN package (4) (version 2.11.1). We created a Phyloseq object for subsequent microbial analyses with the Phyloseq (28) package (version 1.23.1) and we discarded samples of with less than 1,000 reads, with no assignment at the phylum level, ASVs with an average relative abundance <1e-5, and ASVs that were not observed more than 2 times in at least 10% of the samples. We normalized our samples using the negative binomial distribution recommended by McMurdie and Holmes (29), implemented in the Bioconductor package DESeq2 (30). Visualizations of microbial relative abundances were carried out in RStudio (version 1.2.1335) and R (version 3.6.1), using the microeco (version 1.1.0) (31) and ampvis2 (version 2.7.34) packages (32). All sequence data was deposited in the NCBI under Bioproject accession number PRJNA1041303.

### Statistical analyses

Statistical analyses were conducted using GraphPad Prism 10 software. Data are presented as mean ± standard error of the mean (SEM). Statistical analyses were performed by two-way ANOVA followed by a *post hoc* Tukey test. Microbiota relative abundance was assessed using Kruskal Wallis and β-diversity using Permutational Multivariate Analysis of Variance (PERMANOVA). Differences between means were statistically significant, with a P-value <0.05.

## Results

### HMPV viral load is not detected in the intestine of HMPV-infected mice

**Figure 1:**
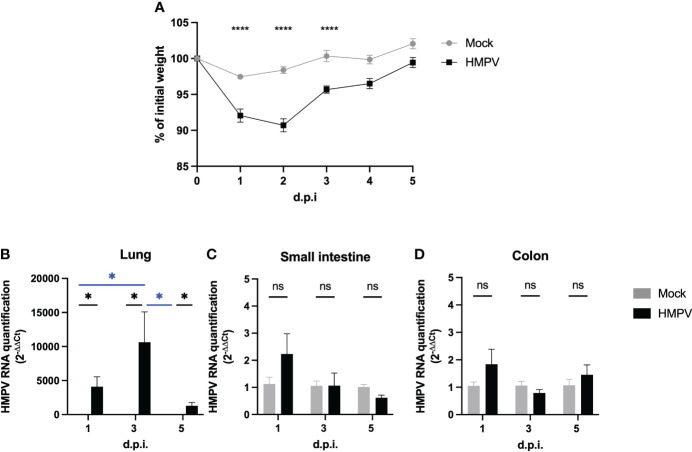
HMPV-infected mice do not exhibit viral load in the intestine at different post-infection times. C57BL/6 mice were infected for 1, 3, and 5 days with HMPV and compared to mice treated with mock. **(A)** Mouse weight was assessed at different time points of the infection and compared to their initial weight. **(B-D)** HMPV viral load was assessed using RT-qPCR at different time points post-infection (days 1, 3, and 5) in the lung **(B)**, small intestine **(C)**, and colon **(D)**. Viral load was assessed by comparing to the expression of a host housekeeping gene (β-actin). Mock-treated mice are shown in grey, and HMPV-infected mice are shown in black. All data are shown as mean ± SEM and are representative of two independent experiments (mock-treated mice n=5-6 per group; HMPV-infected mice n=6 per group). Statistical differences were evaluated by a two-way ANOVA comparing the means of all the columns and rows corresponding to each group, followed by a *post hoc* Tukey test (*p<0.05, ****p<0.0001). ns, not significant.

To assess the effect of HMPV infection in the intestinal tract, we infected intranasally C57BL/6 mice for 1, 3, or 5 days and compared them to mice treated with mock supernatant. Mouse weight was monitored throughout the experiment, and we observed a significant weight loss at days 1 to 3 post-infection (**Figure 1A**). Lung inflammation was confirmed by increased recruitment of neutrophils and monocyte-derived macrophages (**Supplementary Figure 1**). Infection was confirmed by assessing the HMPV viral load in the lung, which was significantly higher at all time points evaluated as compared to the mock-treated group (**Figure 1B**). In contrast, we did not detect the virus in infected mice’s small intestine or colon (**Figures 1C, D**), suggesting that the virus does not infect or replicate in the intestinal tract.

### HMPV-infected mice exhibit expression of IFN-γ in the colon

**Figure 2:**
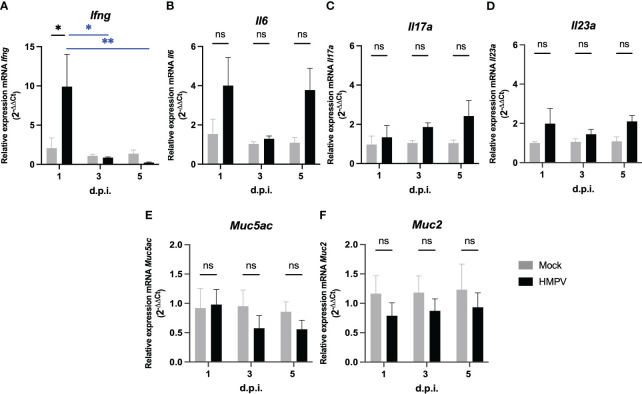
HMPV-infected mice exhibit increased expression of IFN-γ in the colon at day 1 post-infection. Transcription of different pro-inflammatory cytokines and mucins was assessed in the colon of mock-treated and HMPV-infected mice at different time points. **(A)** Relative expression of *Ifng*. **(B)** Relative expression of *Il17a*. **(C)** Relative expression of *Il6*. **(D)** Relative expression of *Il23a.* **(E)** Relative expression of *Muc5ac*. **(F)** Relative expression of *Muc2*. Mock-treated mice are shown in grey, and HMPV-infected mice are shown in black. All data are shown as mean ± SEM and are representative of two independent experiments (mock-treated mice n=5-6 per group; HMPV-infected mice n=6 per group). Statistical differences were evaluated by a two-way ANOVA comparing the means of all the columns and rows corresponding to each group, followed by a *post hoc* Tukey test (*p<0.05 and **p<0.01). ns, not significant.

Next, we assessed whether HMPV-infected exhibit altered levels of cytokine production in the intestinal tract at the RNA level. We analyzed the transcription of the pro-inflammatory cytokines *Ifng*, *Il6*, *Il17a*, and *Il23a*, which are important signals secreted by different innate cells in the onset of intestinal inflammation. Increased expression of IFN-γ, IL-6, IL17-A but not IL-23 has been reported in the intestine of mice infected with influenza virus (2), and therefore we explored whether similar cytokine expression patterns could be observed following HMPV infection. We observed that *Ifng* was significantly increased at day 1 post-infection (**Figure 2A**), whereas we observed a not significant increase of *Il6* at day 1 and 5 post-infection (**Figure 2B**). We did not observe significant differences in *Il17a* and *IL23a* transcription in the colon (**Figures 2C, D**) and none of the cytokines evaluated in the small intestine, when comparing mock-treated and HMPV-infected mice (**Supplementary Figure 2A-D**). However, we observed a significant difference in *Il6* transcription in the small intestine at day 1 post-infection, compared to HMPV-infected mice at day 3 and 5 (**Supplementary Figure 2B**). These results suggest that HMPV may induce increased IFN-γ expression at early infection but none of the other inflammatory cytokines analyzed are increased following HMPV infection.

On the other hand, considering previous work showing increased production of the gastric mucin Muc5ac and the antimicrobial peptide lipocalin-2 following HRSV infection (5), we analyzed the transcription of Muc5ac and the predominant intestinal mucin Muc2 at the RNA level and presence of lipocalin-2 protein in feces. We did not detect significant differences in the transcription of Muc5ac and Muc2 in the colon (**Figures 2E, F**) or small intestine (**Supplementary Figure 2E, F**) following HMPV infection. In addition, the expression of the antimicrobial peptides RegIIIγ (*RegIIIg*) and lipocalin-2 (*Lcn2*) was not altered following HMPV infection in either the small intestine or colon at the RNA level and protein level in fecal supernatants (**Supplementary Figure 3**). These results suggest that mucin expression and antimicrobial peptide expression is not altered following HMPV infection.

### HMPV-infected mice exhibit altered frequency of monocytes in the colon

**Figure 3:**
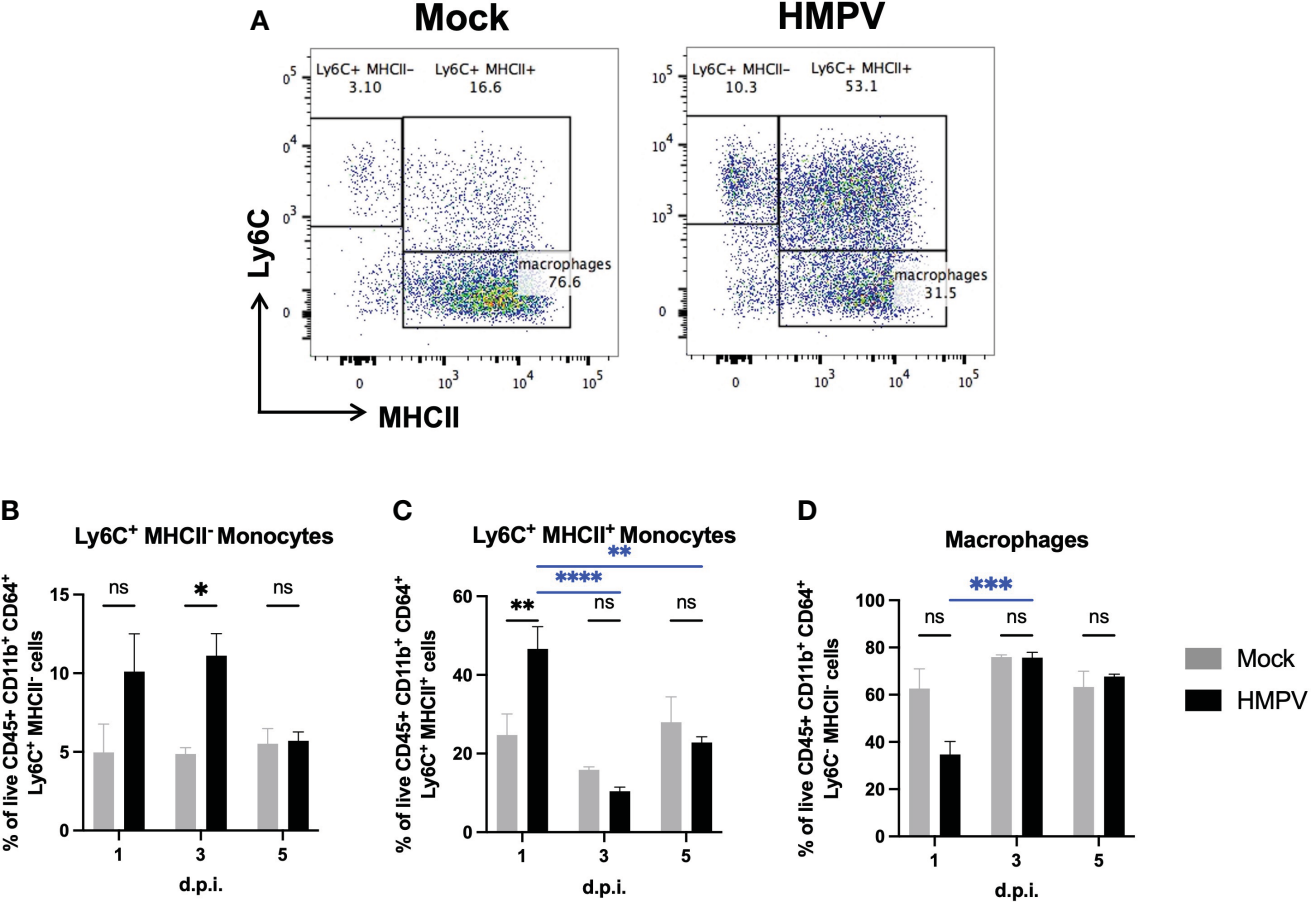
HMPV-infected mice exhibit increased frequency of proinflammatory monocytes in the colon at early infection. Colon lamina propria cells were analyzed at different time points following HMPV infection using flow cytometry. **(A)** Representative plots of colonic Ly6C^+^ MHCII^-^, Ly6C^+^ MHCII^+^, and macrophages (gated as live CD45^+^ CD11b^+^ Ly6G^-^ SiglecF^-^ CD64^+^ cells) in mock-treated and HMPV-infected mice at day 5 post-infection. **(B)** Frequency of Ly6C^+^ MHCII^-^ monocytes. **(C)** Frequency of Ly6C^+^ MHCII^+^ monocytes. **(D)** Frequency of macrophages. Mock-treated mice are shown in grey, and hMPV-infected mice are shown in black. All data are shown as mean ± SEM and are representative of two independent experiments (mock-treated mice n=5-6 per group; HMPV-infected mice n=6 per group). Statistical differences were evaluated by a two-way ANOVA comparing the means of all the columns and rows corresponding to each group, followed by a *post hoc* Tukey test (*p<0.05, **p<0.01, ***p<0.001, ****p<0.0001). ns, not significant.

To identify potential alterations associated with inflammation in the colon, we isolated lamina propria colonic cells and analyzed the frequency of the myeloid population using flow cytometry. Monocytes constantly enter the lamina propria from the circulation and replenish the intestinal macrophage pool, but their recruitment is increased in response to inflammatory signals (33, 34). We analyzed monocyte populations based on the expression of Ly6C^+^ and MHCII^+^, using the monocyte “waterfall” gating strategy (33, 34), as increased frequency of double-positive Ly6C^+^ MHCII^+^ monocytes into the colon lamina propria have been associated with the onset of intestinal inflammation (33, 34) (**Supplementary Figure 4A**). Ly6C^+^ MHCII^-^ monocytes represent a classical phenotype of monocytes freshly recruited from the circulation into the gut. Therefore, if respiratory viral infection induces monocyte recruitment into the colon lamina propria, it would be expected to find increased frequency of classical Ly6C^+^ MHCII^-^ and Ly6C^+^ MHCII^+^ monocytes. We found a significantly increased frequency of Ly6C^+^ MHCII^-^ monocytes at day 3 post-infection, but its frequency was not significantly different compared to the mock-treated group at day 5 post-infection (**Figures 3A, B**). In line with this, we observed a significant increase in double positive Ly6C^+^ MHCII^+^ monocytes at day 1 post-infection (**Figures 3A, C**). However, the numbers of these monocytes were not significantly different at days 3 and 5 post-infection (**Figures 3A, C**), suggesting that this may reflect a transient differentiation towards a pro-inflammatory monocyte phenotype at early infection (**Figures 3A, C**). We did not find significant alterations in the frequency of macrophages (**Figure 3D**) or neutrophils (**Supplementary Figure 4B, C**) at any time point analyzed. These results suggest that HMPV infection may induce transient recruitment of monocytes into the gut at early infection.

### HMPV alters the frequency of colonic CD8^+^ T cells associated with a memory phenotype but not IgA production at day 5 post-infection

**Figure 4:**
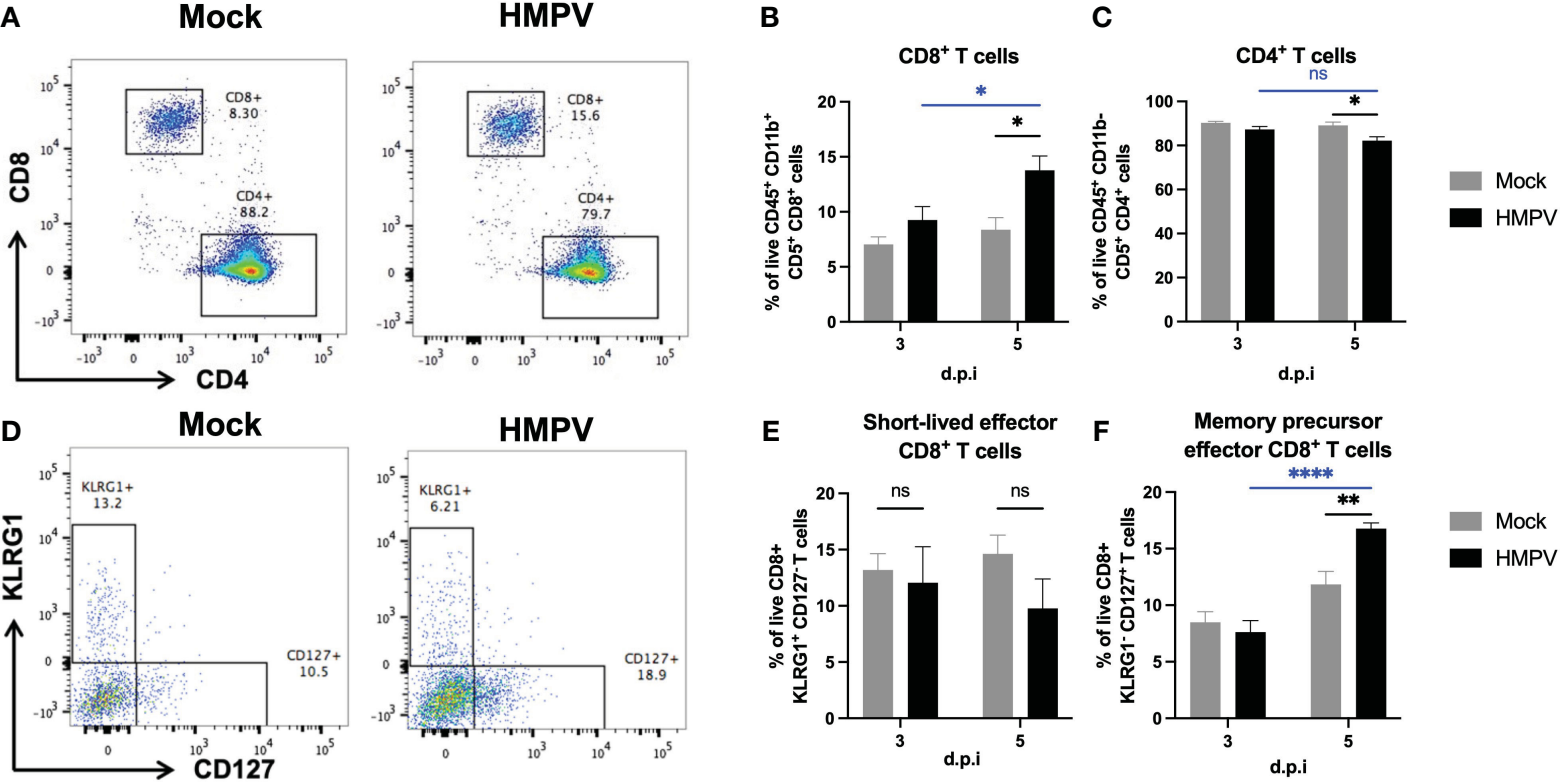
HMPV-infected mice exhibit increased frequency of CD8^+^ T cells in the colon at day 5 post-infection. Colon lamina propria T cells were analyzed at different time points following HMPV infection using flow cytometry. **(A)** Representative plots of CD8^+^ and CD4^+^ T cells (gated as live CD45^+^ CD11b^-^ CD5^+^ cells) in Mock and HMPV-infected mice at day 5 post-infection. **(B)** Frequency of CD8^+^ T cells. **(C)** Frequency of CD4^+^ T cells. **(D)** Representative plots of memory precursor effector CD8+ T cells (identified as KLRG1^-^ CD127^+^ cells) and short-lived effector memory CD8+ T cells (identified as KLRG1^+^ CD127^-^ cells). **(E)** Frequency of short-lived effector CD8^+^ T cells. **(F)** Frequency of memory precursor effector CD8^+^ T cells. Mock-treated mice are shown in grey, and HMPV-infected mice are shown in black. All data are shown as mean ± SEM and are representative of two independent experiments (mock-treated mice n=5-6 per group; HMPV-infected mice n=6 per group). Statistical differences were evaluated by a two-way ANOVA comparing the means of all the columns and rows corresponding to each group, followed by a *post hoc* Tukey test (*p<0.05, **p<0.01, ****p<0.0001). ns, not significant.

Further, we analyzed whether the frequency of adaptive immune cells was altered following HMPV infection. Following influenza infection, the induction of intestinal effector CD8^+^ T cells has been reported (35). We analyzed changes in the frequency of CD8^+^ T cells in the colon using flow cytometry at later infection timepoints (day 3 and day 5). We observed an increased frequency of these cells at day 5 in relation to the frequency of CD4^+^ T cells (**Figures 4A-C**). We also used the surface markers KLRG1 and CD127 to identify memory precursor effector CD8^+^ T cells (KLRG1^-^ CD127^+^) and short-lived effector CD8^+^ T cells (KLRG1^+^ CD127^-^). We found a significant increase in the frequency of memory precursor effector CD8^+^ T cells (**Figures 4D–F**), suggesting that HMPV infection may induce changes in adaptive intestinal immunity. In addition, we evaluated whether these effects are maintained 7 days post-infection and we only found increased frequency of colonic CD8^+^ T cells but no differences in colonic neutrophil and monocyte populations (**Supplementary Figure 5**), suggesting that colonic CD8^+^ T cells may be maintained following respiratory HMPV infection.

**Figure 5:**
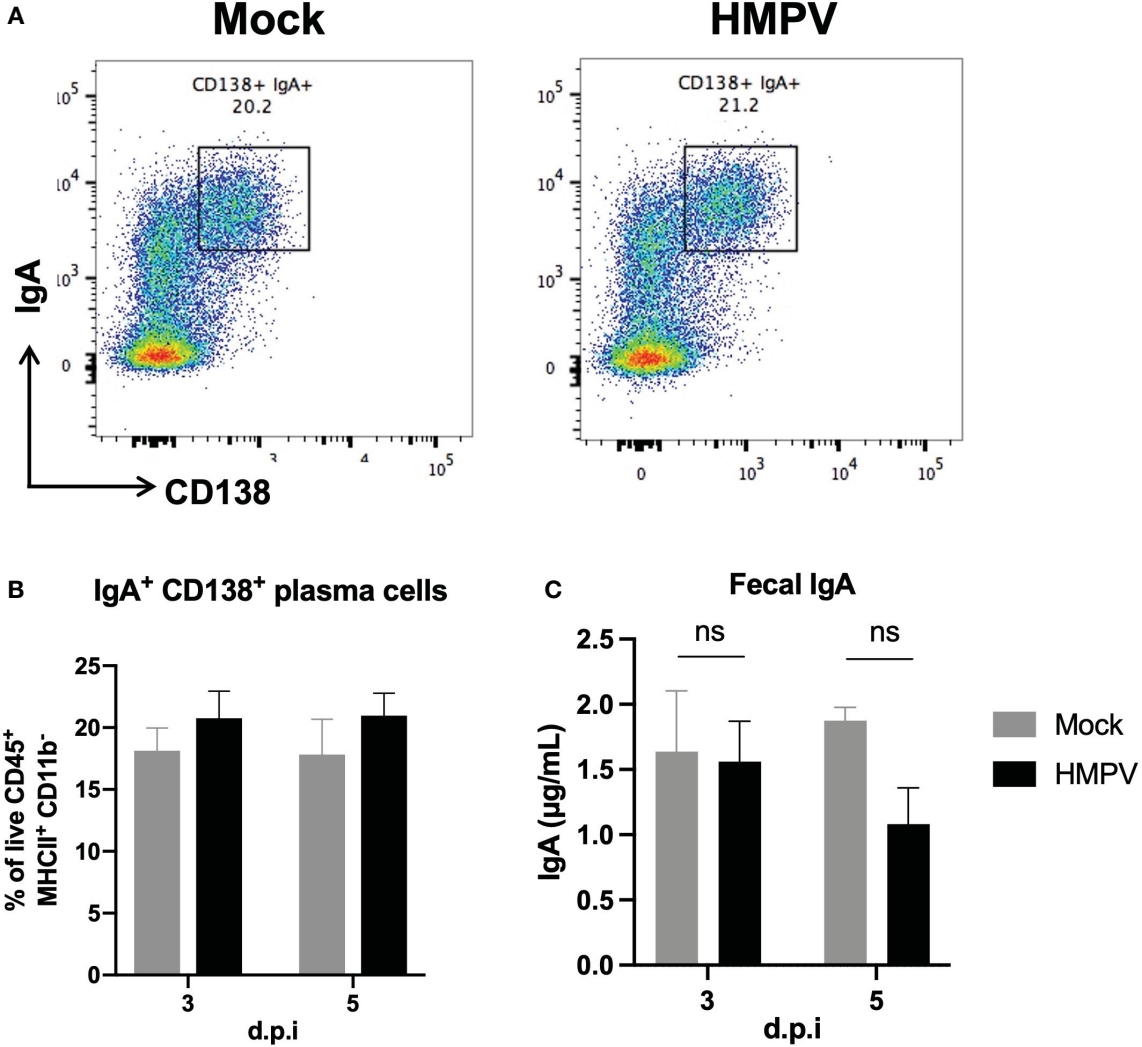
Colonic IgA production is not altered following HMPV infection. IgA production was assessed using flow cytometry and ELISA following HMPV infection. **(A)** Representative plots of colonic IgA^+^ plasma cells (gated as live CD45^+^ MHCII^+^ CD11b^-^ cells) in mock-treated and HMPV-infected mice at day 5 post-infection. **(B)** Frequency of colonic IgA^+^ plasma cells. **(C)** Evaluation of the concentration of free IgA measured by ELISA in feces isolated from HMPV-infected mice at different post-infection times. Mock-treated mice are shown in grey, and HMPV-infected mice are shown in black. All data are shown as mean ± SEM and are representative of two independent experiments (mock-treated mice n=5-6 per group; HMPV-infected mice n=6 per group). Statistical differences were evaluated by a two-way ANOVA comparing the means of all the columns and rows corresponding to each group, followed by a *post hoc* Tukey test. ns, not significant.

Further, considering previous work has shown alterations in antimicrobial responses following viral infection (5), we analyzed whether IgA was altered following HMPV infection. Colonic IgA plasma cells are responsible for IgA secretion and can control the intestinal microbiota composition (36–38). We analyzed changes in the frequency of IgA+ plasma cells (CD138^+^ IgA^+^) in the colon using flow cytometry at late infection timepoints (day 3 and day 5). We did not observe significant differences in the frequency of these cells between mock-treated and HMPV-infected mice (**Figures 5A, B**). We also evaluated total fecal IgA production using ELISA and observed a slight but non-significant decrease in IgA production at day 5 post-infection (**Figure 5C**). Similarly, we did not find significant differences in fecal lipocalin-2 and IgA production 7 days post-infection (**Supplementary Figure 5**). These results indicate that intestinal anti-microbial IgA responses are not altered following HMPV infection.

### HMPV infection does not significantly alter the fecal microbiota composition

**Figure 6:**
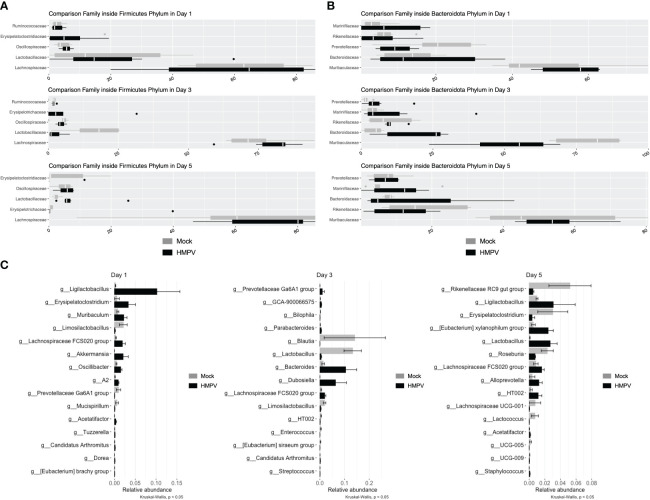
HMPV-infected mice do not exhibit significant alterations in the fecal microbiota. Bacterial DNA from fecal samples was isolated, and 16S rRNA sequencing was performed. Mock and HMPV microbiota were grouped by day post-infection. **(A)** Microbiota abundance between family members of the phylum Firmicutes. **(B)** Microbiota abundance between family members of the phylum Bacteroidota. **(C)** Microbiota abundance at the genus level, showing the 15 most abundant genera at each time point (Kruskal-Wallis, p < 0.05). Mock-treated mice are shown in grey, and HMPV-infected mice are shown in black. All data are shown as mean ± SD and are representative of two independent experiments (mock-treated mice n=5-6 per group; HMPV-infected mice n=6 per group).

Several reports have shown that respiratory viral infection may alter the composition of the intestinal microbiota and even favor the expansion of pathogenic species (2, 4–6). We analyzed the fecal microbiota of mock-treated and HMPV-infected mice using 16S rRNA sequencing. Relative abundance of members of the Firmicutes and Bacteroidota phylum did not exhibit significant differences at any time point (**Figures 6A, B**), although we observed a non-significant decrease the family *Lactobacillaceae* at day 3, but its relative abundance was similar between mock-treated and HMPV-treated mice by day 5 (**Figure 6A**). In line with this, relative abundance was not significantly different between bacterial genera, but we observed a non-significant an increase in *Ligilactobacillus* (**Figure 6C**). Alpha diversity was also analyzed (Shannon and Observed) but no significant differences were detected (**Supplementary Figure 6A**) whereas beta-diversity was not significantly different between mock-treated and HMPV-infected mice at each time point (**Supplementary Figure 6B**). Of note, the fecal microbiota of the analyzed mice corresponds to those euthanized at days 1, 3, and 5 post-infection, respectively. These results suggest that although HMPV may slightly alter the fecal microbiota composition at early time points post-infection, mice do not exhibit statistically significant differences in the fecal microbiota composition, without expansion of pathogenic species.

## Discussion

The effect of respiratory viruses on intestinal immunity is an area of increasing interest due to the potential increase in susceptibility to both intestinal and systemic disease following viral infection (39). To our knowledge, this is the first report describing the alterations in intestinal immunity following respiratory infection with HMPV infection. These effects do not seem to be mediated by direct viral infection in the intestinal tract. We confirmed HMPV viral load in the lungs post-infection at each time point, but no viral load was detected in the small intestine and colon (**Figure 1**). Our results suggest that the virus does not replicate in the intestinal tract, but we cannot rule out that in our studies, some of the virus administered intranasally may have accessed the oral route during the infection, which may have contributed to the alterations observed in day 1 post-infection. The main known target of HMPV are airway epithelial cells (AECs), through the interaction between the viral fusion protein F and heparan sulfate on the surface of AECs and thus HMPV infection has been mainly associated to the airways (40, 41). Although intestinal epithelial cells also express heparan sulfate (42), it is not known whether the virus is also able to target these cells and/or whether the virus is able to reach the intestinal epithelial considering the presence of the mucus layer present in both small and large intestine. Other respiratory viruses such as SARS-CoV-2 have been shown to infect enterocytes and viral load has been detected in intestinal biopsies and the stools of patients exhibiting diarrhea (43–45). Considering the lack of detection of HMPV in intestinal tissue in our experiments, our results align with those reported for murine infection with influenza A and HRSV, as there is no direct infection in the gut, yet there is a change in the intestinal immune response and microbiota composition (2, 4–6). Indeed, intragastric administration of influenza A does not cause infection and intestinal inflammation in a murine infection model (2). Therefore, *in vitro* assays assessing HMPV ability to infect intestinal epithelial cell lines and/or *in vivo* intragastric administration of the virus could be performed to elucidate whether early alterations in the gut upon HMPV infection are due to direct viral infection into the intestine or to indirect distal effects. However, here we report changes in the intestinal immunity that are observed up to day 5 post-infection, so it is unlikely that they are mediated directly by HMPV presence in the intestine.

The relative expression of various proinflammatory cytokines was measured to assess whether HMPV caused an inflammatory response in the intestine. We observed increased expression of IFN-γ at day 1 post-infection but no major changes in other cytokines at later timepoints post-infection in either the small intestine or colon, suggesting that HMPV respiratory infection may alter innate immunity in the colon. Although we did not assess the cellular source of this cytokine, several local and recruited innate cells could be responsible for these effects, including neutrophils and NK cells (46). On the other hand, IFN-γ also promotes inflammatory monocyte recruitment in other infections. However, other signals, including the chemokine CCL2, have been shown to play an important role in monocyte recruitment during intestinal inflammation (47, 48). IL-23 can also promote monocyte recruitment (47), but we did not find a significant increase in the expression of this cytokine following HMPV infection in the intestine. As reported by flow cytometry, we found an altered frequency of monocytes infiltrating the colon at early infection. It has also been described that during colitis induced in C57BL/6 mice, increased recruitment of Ly6C^+^ MHCII^-^ monocytes results in increased frequency of Ly6C^+^ MHCII^+^ antigen-presenting monocytes, which play pro-inflammatory roles (33, 49). Our results show that both IFN-γ expression and increased frequency of monocytes occurs transiently at early infection. Still, normal levels are restored by day 5, suggesting that HMPV infection does not profoundly alter these parameters in intestinal immunity. However, a limitation of our study is the lack of measurement of protein levels, including IFN-γ, in intestinal lavage or tissue, as reported for the effects of HRSV infection in intestinal tissue (5), which could further confirm our findings.

On the other hand, we evaluated the frequency of CD8^+^ T cells and CD8^+^ T cell subsets and found an increased frequency of memory precursor effector CD8^+^ T cells (**Figure 4**). It has been reported that these cells expressing the IL-7 receptor, CD127 can give rise to long-lived memory cells, and their fate is regulated by IL-15 and low expression of T-bet, compared to KLRG1^+^ short-lived effector cells, whose fate relies on IL-12 signaling to drive high expression of T-bet (50, 51). Importantly, a previous study showed that antigen-specific KLRG1^+^ short-lived effector cells but not memory precursor effector CD8^+^ T cells are increased in the intestinal lamina propria 10 days after intranasal infection with influenza virus expressing ovalbumin, suggesting that respiratory viruses may have differential effects on CD8^+^ T cell intestinal subsets (35). These changes in intestinal CD8^+^ T cell subsets were attributed to the migration of these cells into the intestinal mucosa, which could be an explanation for the increase in both intestinal CD8^+^ T cells and memory precursor effector CD8^+^ T cells reported here in response to HMPV infection. A similar case has been described for influenza A infection, which drives lung CD4^+^ T cell migration with increased gut homing, which causes Th17-dependent intestinal inflammation influenced by the microbiota (2). However, a limitation of our study was the lack of characterization of antigen-specific CD8^+^ T cell responses, which could confirm whether these intestinal CD8^+^ T cells are specific against HMPV. In addition, we did not perform histopathology in colon samples, which could have confirmed the recruitment of monocytes and CD8^+^ T cells at the different time points indicated by flow cytometry.

Different secreted proteins, including innate cell-secreted antimicrobial peptides and plasma cell-secreted IgA, regulate intestinal antimicrobial responses and microbiota composition. HRSV infection has been previously shown to cause an increase in the expression of fecal lipocalin-2, which was also associated with low-grade colon inflammation 7 days post-infection (5). Neutrophils constitutively express lipocalin-2 and increased fecal levels have been proposed as a sensitive marker of gut dysbiosis and intestinal inflammation in mouse models of colitis (52–54). Here, we did not find alterations in the expression of lipocalin-2 and the antibacterial lectin RegIIIγ in both the small intestine and colon at the RNA level (**Supplementary Figure 4**) and fecal supernatants (**Figure 2F**), in contrast to the results reported during HRSV infection (5), suggesting that HMPV infection does not alter innate antimicrobial responses. These results are consistent with the lack of significant differences in neutrophil recruitment to the colon of HMPV-infected mice, which suggests that HMPV does not induce neutrophil-mediated colonic inflammation (**Supplementary Figure 4C**). In line with this, we also did not find changes in the expression of the gastric Muc5ac in the intestine, which has been reported to be increased following HRSV infection (5). Similarly, we assessed the frequency of IgA^+^ plasma cells and levels of fecal IgA (**Figure 5**), as levels of microbiota-specific IgA can change in response to infection and inflammation in the intestine, driving alterations in the microbiota composition (36, 38). Our results indicate that levels of IgA are not altered following HMPV, which is consistent with the lack of significant variations in the fecal microbiota. However, we did not evaluate the generation of HMPV-specific antibodies in the intestinal tract, which could be explored in further studies.

Changes in the intestinal microbiota during respiratory infections have been extensively studied through the lung-gut axis (39). To assess whether HMPV infection caused changes in the intestinal microbiota, the relative abundance of different bacterial groups characteristic of the murine normal microbiota was analyzed using 16 rRNA sequencing. We found alterations in the relative abundance of some of the most abundant genera in our samples. However, we did not find significant differences in particular genera and/or distinctive microbiota in HMPV-infected mice, and we did not find alterations in β-diversity, which is in line with the lack of alteration in molecules related to antimicrobial immunity. Various studies have shown changes in the intestinal microbiota during HRSV infection (5, 6), which belongs to the same taxonomic family as HMPV. HRSV-induced changes in the intestinal microbiota composition occur due to alterations in food intake caused by CD8^+^ T cell-dependent inappetence and a decrease in food intake (6). In addition, it was reported that HRSV induces changes in the fecal metabolome, altering lipid metabolism (6). Although both HRSV and HMPV belong to the family *Pneumoviridae* (55), we report that hMPV does not cause a profound and lasting effect on the intestinal microbiota. Importantly, we did not find an expansion of potentially pathogenic bacteria belonging to the *Enterobacteriaceae* family, which are increased during influenza A infection in mice and are responsible for the induction of microbiota-dependent intestinal inflammation (2). Of note, we did not compare the fecal microbiota before and after the infection but all animals used in this study were littermates that were kept in identical environmental conditions. These findings suggest that the immune changes reported here are not attributed to local changes in the intestinal microbiota but reflect indirect effects of respiratory virus, such as inappetence observed in early infection.

Taken together, our data indicates that HMPV does not cause extensive inflammation in the colon but rather induces transient recruitment of monocytes at early infection and increased frequency of CD8^+^ T cells, with the potential of becoming memory cells at late infection. Of note, we used C57BL/6 mice as a model of infection and has been reported as a murine model of acute, self-resolving infection of HMPV, which do not develop extensive lung damage and are able to clear the virus in 10 days (56, 57). In line with this, we analyzed whether mice exhibit differences in colon lamina propria cells by flow cytometry and we only confirmed an increase in CD8^+^ T cells but no differences in monocyte and neutrophil recruitment, which suggests that CD8^+^ T cell increase is maintained at later time points following HMPV but it is not necessarily related with colon inflammation. We cannot rule out that models with higher susceptibility to HMPV, such as BALB/c mice (57) or mouse models susceptible to spontaneous inflammation, such as IL-10^-/-^ mice, may exhibit more pronounced intestinal inflammation and changes in microbiota composition (38, 58). On the other hand, changes in intestinal immunity caused by influenza infection render increased susceptibility to *Salmonella enterica* serovar Typhimurium secondary infection in mouse models. In contrast, it is unclear whether HMPV may alter susceptibility to secondary intestinal pathology (3, 59). Therefore, further studies are required to understand the impact of HMPV respiratory infection on the susceptibility to secondary intestinal infection or inflammation.

## Data availability statement

The original contributions presented in the study are included in the article/**Supplementary Material**. Further inquiries can be directed to the corresponding authors.

## Ethics statement

The animal study was approved by Comité Ético Científico para el Cuidado de Animales y Ambiente, Pontificia Universidad Católica de Chile; Comité de Bioética, Universidad Andrés Bello. The study was conducted in accordance with the local legislation and institutional requirements.

## Author contributions

JS-A: Conceptualization, Data curation, Investigation, Writing – original draft, Writing – review & editing. EC: Data curation, Investigation, Writing – review & editing. OV: Data curation, Investigation, Writing – review & editing. IR-T: Data curation, Software, Writing – original draft, Writing – review & editing. CM-M: Data curation, Software, Writing – original draft, Writing – review & editing. MM-L: Data curation, Investigation, Writing – original draft, Writing – review & editing. IS: Data curation, Investigation, Writing – review & editing. ER-A: Data curation, Investigation, Writing – review & editing. PS: Investigation, Writing – review & editing. OA-M: Investigation, Writing – review & editing. DC-G: Investigation, Writing – review & editing. CR: Writing – review & editing. KS: Writing – review & editing. JU: Writing – review & editing. JS: Writing – review & editing. SB: Conceptualization, Writing – review & editing. AK: Conceptualization, Writing – review & editing. FM-G: Conceptualization, Data curation, Project administration, Supervision, Writing – original draft, Writing – review & editing.

## Supplementary material

Supplementary Figure 1 HMPV-infected mice exhibit increased recruitment of lung neutrophils and monocyte-derived macrophages. Lung cells were analyzed at different time points following HMPV infection using flow cytometry. A) Representative plots of neutrophils (gated as live CD45+ CD11b+ Ly6G+ cells) in Mock and HMPV-infected mice at day 1 post-infection. B) Frequency of neutrophils. C) Representative plots of monocyte-derived macrophages (gated as live CD45+ CD11b+ SiglecF- MHCII+ Ly6C+ CD64+ cells) in Mock and HMPV-infected mice at day 1 post-infection. D) Frequency of monocyte-derived macrophages. Mock-treated mice are shown in grey, and HMPV-infected mice are shown in black. All data are shown as mean ± SEM and are representative of two independent experiments (mock-treated mice n=5-6 per group; HMPV-infected mice n=6 per group). Statistical differences were evaluated by a two-way ANOVA comparing the means of all the columns and rows corresponding to each group, followed by a post hoc Tukey test (*p<0.05 and **p<0.01).

Supplementary Figure 2 HMPV-infected mice do not exhibit changes in pro-inflammatory cytokines in the small intestine. Transcription of different pro-inflammatory cytokines was assessed in the small intestine of mock-treated and HMPV-infected mice at different time points. A) Relative expression of Ifng. B) Relative expression of Il6. C) Relative expression of Il17a. D) Relative expression of Il23a. E) Relative expression of Muc5ac. F) Relative expression of Muc2. Data representative of two independent experiments (mock-treated mice n=5-6 per group; HMPV-infected mice n=6 per group). Mock-treated mice are shown in grey, and HMPV-infected mice are shown in black. All data are shown as mean ± SEM and are representative of two independent experiments (mock-treated mice n=5-6 per group; HMPV-infected mice n=6 per group). Statistical differences were evaluated by a two-way ANOVA comparing the means of all the columns and rows corresponding to each group, followed by a post hoc Tukey test (*p<0.05 and **p<0.01).

Supplementary Figure 3 HMPV-infected mice do not exhibit changes in the transcription of antimicrobial peptides in the small intestine and colon and fecal levels of Lipocalin-2. Expression at the RNA level of different pro-inflammatory cytokines was assessed in the small intestine and colon of mock-treated and HMPV-infected mice at different time points. A) Relative expression of RegIIIg in the small intestine. B) Relative expression of RegIIIg in the colon. C) Relative expression of Lcn2 in the small intestine. D) Relative expression of Lcn2 in the colon. E) Fold change of fecal lipocalin-2 levels measured by ELISA in fecal supernatants. Mock-treated mice are shown in grey, and HMPV-infected mice are shown in black. All data are shown as mean ± SEM and are representative of two independent experiments (mock-treated mice n=5-6 per group; HMPV-infected mice n=6 per group). Statistical differences were evaluated by a two-way ANOVA comparing the means of all the columns and rows corresponding to each group, followed by a post hoc Tukey test.

Supplementary Figure 4 HMPV-infected mice do not exhibit increased recruitment of neutrophils into the colon at different post-infection time points. Colon lamina propria cells were analyzed at different time points following HMPV infection using flow cytometry. A) Gating strategy for neutrophils (live CD45+ CD11b+ Ly6G+ cells), macrophages (live CD45+ CD11b+ CD64+ Ly6C- MHCII+ cells) and monocytes (live CD45+ CD11b+ CD64+ Ly6C+ MHCII-/+ cells). B) Representative plots of neutrophils (gated as live CD45+ CD11b+ Ly6G+ cells) in Mock and HMPV-infected mice at day 1 post-infection. C) Frequency of colonic neutrophils. Mock-treated mice are shown in grey, and HMPV-infected mice are shown in black. All data are shown as mean ± SEM and are representative of two independent experiments (mock-treated mice n=5-6 per group; HMPV-infected mice n=6 per group). Statistical differences were evaluated by a two-way ANOVA comparing the means of all the columns and rows corresponding to each group, followed by a post hoc Tukey test.

Supplementary Figure 5 HMPV-infected mice do not exhibit changes in the expression of antimicrobial peptides in the small intestine and colon. Colon lamina propria cells were analyzed at different time points following HMPV infection using flow cytometry. A) Frequency of colonic neutrophils. B) Frequency of colonic monocytes and macrophages. C) Frequency of CD8+ T cells. Expression of lipocalin-2 and IgA was measured in fecal supernatants by ELISA. D) Fold change of fecal lipocalin-2 levels. E) Levels of free fecal IgA. Mock-treated mice are shown in grey, and HMPV-infected mice are shown in black. All data are shown as mean ± SEM and are representative of two independent experiments (mock-treated mice n=5 per group; HMPV-infected mice n=6 per group). Statistical differences were evaluated using an unpaired t test.

Supplementary Figure 6 HMPV-infected mice do not exhibit fecal microbiota β diversity alterations. A) Alpha diversity of fecal Mock and HMPV samples at different timepoints, analyzed by Observed and Shannon Index. Mock-treated mice are shown in grey, and HMPV-infected mice are shown in black. B) PCoA analysis of all samples by group (mock and HMPV) and timepoint. Mock samples are shown as triangles, and HMPV samples are shown as circles. Time points are shown in green (day 1), red (day 3), and blue (day 5). PERMANOVA test was conducted using distance matrices (HMPV samples between days: Unifrac, Jaccard y Bray-Curtis, HMPV, p < 0.05; Mock samples between days: Unifrac (p < 0.001); wUnifrac (p < 0.05); Bray- Curtis (p < 0.001) and Jaccard (p < 0.001); no significant differences between mock and HMPV samples at any timepoint). Data are representative of two independent experiments (mock-treated mice n=5-6 per group; HMPV-infected mice n=6 per group).

## References

[1] Torres A Cilloniz C Niederman MS Menéndez R Chalmers JD Wunderink RG. Pneumonia. Nat Rev Dis Primers (2021) 7:1–28. doi: 10.1038/s41572-021-00259-0 33833230

[2] Wang J Li F Wei H Lian Z-X Sun R Tian Z. Respiratory influenza virus infection induces intestinal immune injury *via* microbiota-mediated Th17 cell-dependent inflammation. J Exp Med (2014) 211:2397–410. doi: 10.1084/jem.20140625 PMC4235643 25366965

[3] Deriu E Boxx GM He X Pan C Benavidez SD Cen L. Influenza virus affects intestinal microbiota and secondary salmonella infection in the gut through type I interferons. PloS Pathog (2016) 12:e1005572. doi: 10.1371/journal.ppat.1005572 27149619 PMC4858270

[4] Yildiz S Mazel-Sanchez B Kandasamy M Manicassamy B Schmolke M. Influenza A virus infection impacts systemic microbiota dynamics and causes quantitative enteric dysbiosis. Microbiome (2018) 6:9. doi: 10.1186/s40168-017-0386-z 29321057 PMC5763955

[5] Groves HT Cuthbertson L James P Moffatt MF Cox MJ Tregoning JS. Respiratory disease following viral lung infection alters the murine gut microbiota. Front Immunol (2018) 9:182. doi: 10.3389/fimmu.2018.00182 29483910 PMC5816042

[6] Groves HT Higham SL Moffatt MF Cox MJ Tregoning JS. Respiratory viral infection alters the gut microbiota by inducing inappetence. mBio (2020) 11:e03236–19. doi: 10.1128/mBio.03236-19 PMC7029140 32071269

[7] van den Hoogen BG de Jong JC Groen J Kuiken T de Groot R Fouchier RA. A newly discovered human pneumovirus isolated from young children with respiratory tract disease. Nat Med (2001) 7:719–24. doi: 10.1038/89098 PMC7095854 11385510

[8] Lefebvre A Manoha C Bour J-B Abbas R Fournel I Tiv M. Human metapneumovirus in patients hospitalized with acute respiratory infections: A meta-analysis. J Clin Virol (2016) 81:68–77. doi: 10.1016/j.jcv.2016.05.015 27337518 PMC7106388

[9] Soto JA Gálvez NMS Benavente FM Pizarro-Ortega MS Lay MK Riedel C. Human metapneumovirus: mechanisms and molecular targets used by the virus to avoid the immune system. Front Immunol (2018) 9:2466. doi: 10.3389/fimmu.2018.02466 30405642 PMC6207598

[10] Lay MK Céspedes PF Palavecino CE León MA Díaz RA Salazar FJ. Human metapneumovirus infection activates the TSLP pathway that drives excessive pulmonary inflammation and viral replication in mice. Eur J Immunol (2015) 45:1680–95. doi: 10.1002/eji.201445021 25763996

[11] Cheemarla NR Baños-Lara M Naidu S Guerrero-Plata A. Neutrophils regulate the lung inflammatory response *via* γδ T cell infiltration in an experimental mouse model of human metapneumovirus infection. J Leukoc Biol (2017) 101:1383–92. doi: 10.1189/jlb.4A1216-519RR PMC5433860 28336678

[12] Kolli D Bataki EL Spetch L Guerrero-Plata A Jewell AM Piedra PA. T lymphocytes contribute to antiviral immunity and pathogenesis in experimental human metapneumovirus infection. J Virol (2008) 82:8560–9. doi: 10.1128/JVI.00699-08 PMC2519650 18562525

[13] Kolli D Gupta MR Sbrana E Velayutham TS Chao H Casola A. Alveolar macrophages contribute to the pathogenesis of human metapneumovirus infection while protecting against respiratory syncytial virus infection. Am J Respir Cell Mol Biol (2014) 51:502–15. doi: 10.1165/rcmb.2013-0414OC PMC4189482 24749674

[14] Diab M Glasner A Isaacson B Bar-On Y Drori Y Yamin R. NK-cell receptors NKp46 and NCR1 control human metapneumovirus infection. Eur J Immunol (2017) 47:692–703. doi: 10.1002/eji.201646756 28191644

[15] Hamelin M-E Prince GA Gomez AM Kinkead R Boivin G. Human metapneumovirus infection induces long-term pulmonary inflammation associated with airway obstruction and hyperresponsiveness in mice. J Infect Dis (2006) 193:1634–42. doi: 10.1086/504262 16703506

[16] Peiris JSM Tang W-H Chan K-H Khong P-L Guan Y Lau Y-L. Children with respiratory disease associated with metapneumovirus in Hong Kong. Emerg Infect Dis (2003) 9:628–33. doi: 10.3201/eid0906.030009 PMC3000155 12781000

[17] Rudd PA Thomas BJ Zaid A MacDonald M Kan-O K Rolph MS. Role of human metapneumovirus and respiratory syncytial virus in asthma exacerbations: where are we now? Clin Sci (Lond) (2017) 131:1713–21. doi: 10.1042/CS20160011 28667069

[18] Boivin G De Serres G Côté S Gilca R Abed Y Rochette L. Human metapneumovirus infections in hospitalized children1. Emerg Infect Dis (2003) 9:634–40. doi: 10.3201/eid0906.030017 PMC3000156 12781001

[19] von Linstow M-L Eugen-Olsen J Koch A Winther TN Westh H Hogh B. Excretion patterns of human metapneumovirus and respiratory syncytial virus among young children. Eur J Med Res (2006) 11:329–35. 17052968

[20] Reina J Ferres F Alcoceba E Mena A de Gopegui ER Figuerola J. Comparison of different cell lines and incubation times in the isolation by the shell vial culture of human metapneumovirus from pediatric respiratory samples. J Clin Virol (2007) 40:46–9. doi: 10.1016/j.jcv.2007.06.006 17644404

[21] Soto JA Gálvez NMS Pacheco GA Canedo-Marroquín G Bueno SM Kalergis AM. Induction of protective immunity by a single low dose of a master cell bank cGMP-rBCG-P vaccine against the human metapneumovirus in mice. Front Cell Infection Microbiol (2021) 11:662714. doi: 10.3389/fcimb.2021.662714 PMC8276701 34268134

[22] Soto JA Gálvez NMS Rivera CA Palavecino CE Céspedes PF Rey-Jurado E. Recombinant BCG vaccines reduce pneumovirus-caused airway pathology by inducing protective humoral immunity. Front Immunol (2018) 9:2875. doi: 10.3389/fimmu.2018.02875 30581437 PMC6293239

[23] Tollefson SJ Cox RG Williams JV. Studies of culture conditions and environmental stability of human metapneumovirus. Virus Res (2010) 151:54–9. doi: 10.1016/j.virusres.2010.03.018 PMC2894476 20380856

[24] Melo-Gonzalez F Hepworth MR. Identification and functional characterization of murine group 3 innate lymphoid cell (ILC3) subsets in the intestinal tract and associated lymphoid tissues. Methods Mol Biol (2020) 2121:37–49. doi: 10.1007/978-1-0716-0338-3_4 32147784

[25] Callahan BJ McMurdie PJ Rosen MJ Han AW Johnson AJA Holmes SP. DADA2: High-resolution sample inference from Illumina amplicon data. Nat Methods (2016) 13:581–3. doi: 10.1038/nmeth.3869 PMC4927377 27214047

[26] Quast C Pruesse E Yilmaz P Gerken J Schweer T Yarza P. The SILVA ribosomal RNA gene database project: improved data processing and web-based tools. Nucleic Acids Res (2013) 41. doi: 10.1093/nar/gks1219 PMC3531112 23193283

[27] Wright ES. RNAconTest: comparing tools for noncoding RNA multiple sequence alignment based on structural consistency. RNA (2020) 26:531–40. doi: 10.1261/rna.073015.119 PMC7161358 32005745

[28] McMurdie PJ Holmes S. phyloseq: an R package for reproducible interactive analysis and graphics of microbiome census data. PloS One (2013) 8:e61217. doi: 10.1371/journal.pone.0061217 23630581 PMC3632530

[29] McMurdie PJ Holmes S. Waste not, want not: why rarefying microbiome data is inadmissible. PloS Comput Biol (2014) 10:e1003531. doi: 10.1371/journal.pcbi.1003531 24699258 PMC3974642

[30] Love MI Huber W Anders S. Moderated estimation of fold change and dispersion for RNA-seq data with DESeq2. Genome Biol (2014) 15:550. doi: 10.1186/s13059-014-0550-8 25516281 PMC4302049

[31] Liu C Cui Y Li X Yao M. microeco: an R package for data mining in microbial community ecology. FEMS Microbiol Ecol (2021) 97:fiaa255. doi: 10.1093/femsec/fiaa255 33332530

[32] Andersen KS Kirkegaard RH Karst SM Albertsen M. ampvis2: an R package to analyse and visualise 16S rRNA amplicon data. Biorxiv (2018), 299537. doi: 10.1101/299537

[33] Bain CC Scott CL Uronen-Hansson H Gudjonsson S Jansson O Grip O. Resident and pro-inflammatory macrophages in the colon represent alternative context-dependent fates of the same Ly6Chi monocyte precursors. Mucosal Immunol (2013) 6:498–510. doi: 10.1038/mi.2012.89 22990622 PMC3629381

[34] Bain CC Bravo-Blas A Scott CL Perdiguero EG Geissmann F Henri S. Constant replenishment from circulating monocytes maintains the macrophage pool in the intestine of adult mice. Nat Immunol (2014) 15:929–37. doi: 10.1038/ni.2967 PMC4169290 25151491

[35] Sheridan BS Pham Q-M Lee Y-T Cauley LS Puddington L Lefrançois L. Oral infection drives a distinct population of intestinal resident memory CD8(+) T cells with enhanced protective function. Immunity (2014) 40:747–57. doi: 10.1016/j.immuni.2014.03.007 PMC4045016 24792910

[36] Pabst O Slack E. IgA and the intestinal microbiota: the importance of being specific. Mucosal Immunol (2020) 13:12–21. doi: 10.1038/s41385-019-0227-4 31740744 PMC6914667

[37] Penny HA Domingues RG Krauss MZ Melo-Gonzalez F Lawson MAE Dickson S. Rhythmicity of intestinal IgA responses confers oscillatory commensal microbiota mutualism. Sci Immunol (2022) 7:eabk2541. doi: 10.1126/sciimmunol.abk2541 36054336 PMC7613662

[38] Melo-Gonzalez F Kammoun H Evren E Dutton EE Papadopoulou M Bradford BM. Antigen-presenting ILC3 regulate T cell-dependent IgA responses to colonic mucosal bacteria. J Exp Med (2019) 216:728–42. doi: 10.1084/jem.20180871 PMC6446868 30814299

[39] Melo-González F Sepúlveda-Alfaro J Schultz BM Suazo ID Boone DL Kalergis AM. Distal consequences of mucosal infections in intestinal and lung inflammation. Front Immunol (2022) 13:877533. doi: 10.3389/fimmu.2022.877533 35572549 PMC9095905

[40] Klimyte EM Smith SE Oreste P Lembo D Dutch RE. Inhibition of human metapneumovirus binding to heparan sulfate blocks infection in human lung cells and airway tissues. J Virol (2016) 90:9237–50. doi: 10.1128/JVI.01362-16 PMC5044844 27489270

[41] Chang A Masante C Buchholz UJ Dutch RE. Human metapneumovirus (HMPV) binding and infection are mediated by interactions between the HMPV fusion protein and heparan sulfate. J Virol (2012) 86:3230–43. doi: 10.1128/JVI.06706-11 PMC3302303 22238303

[42] Yamamoto S Nakase H Matsuura M Honzawa Y Matsumura K Uza N. Heparan sulfate on intestinal epithelial cells plays a critical role in intestinal crypt homeostasis *via* Wnt/β-catenin signaling. Am J Physiol Gastrointest Liver Physiol (2013) 305:G241–9. doi: 10.1152/ajpgi.00480.2012 PMC3742857 23744737

[43] Cheung KS Hung IFN Chan PPY Lung KC Tso E Liu R. Gastrointestinal manifestations of SARS-coV-2 infection and virus load in fecal samples from a Hong Kong cohort: systematic review and meta-analysis. Gastroenterology (2020) 159:81–95. doi: 10.1053/j.gastro.2020.03.065 32251668 PMC7194936

[44] Lin L Jiang X Zhang Z Huang S Zhang Z Fang Z. Gastrointestinal symptoms of 95 cases with SARS-CoV-2 infection. Gut (2020) 69:997–1001. doi: 10.1136/gutjnl-2020-321013 32241899

[45] Lehmann M Allers K Heldt C Meinhardt J Schmidt F Rodriguez-Sillke Y. Human small intestinal infection by SARS-CoV-2 is characterized by a mucosal infiltration with activated CD8+ T cells. Mucosal Immunol (2021) 14:1381–92. doi: 10.1038/s41385-021-00437-z PMC8379580 34420043

[46] Kak G Raza M Tiwari BK. Interferon-gamma (IFN-γ): Exploring its implications in infectious diseases. Biomolecular Concepts (2018) 9:64–79. doi: 10.1515/bmc-2018-0007 29856726

[47] McDermott AJ Falkowski NR McDonald RA Frank CR Pandit CR Young VB. Role of interferon-γ and inflammatory monocytes in driving colonic inflammation during acute Clostridium difficile infection in mice. Immunology (2017) 150:468–77. doi: 10.1111/imm.12700 PMC5343354 27995603

[48] Carneiro MB de Moura Lopes ME Romano A Campos AC Sacks D Vieira LQ. IFN-gamma mediated inflammatory monocyte recruitment neutralizes iNOS-dependent parasite killing by expanding the permissive host cell reservoir during early Leishmania amazonensis infection. J Immunol (2016) 196:135. doi: 10.4049/jimmunol.196.Supp.135.6 26590317

[49] Bain CC Mowat A. Macrophages in intestinal homeostasis and inflammation. Immunol Rev (2014) 260:102–17. doi: 10.1111/imr.12192 PMC4141699 24942685

[50] Joshi NS Cui W Chandele A Lee HK Urso DR Hagman J. Inflammation directs memory precursor and short-lived effector CD8(+) T cell fates *via* the graded expression of T-bet transcription factor. Immunity (2007) 27:281–95. doi: 10.1016/j.immuni.2007.07.010 PMC2034442 17723218

[51] Kaech SM Tan JT Wherry EJ Konieczny BT Surh CD Ahmed R. Selective expression of the interleukin 7 receptor identifies effector CD8 T cells that give rise to long-lived memory cells. Nat Immunol (2003) 4:1191–8. doi: 10.1038/ni1009 14625547

[52] Yadav SK Ito N Mindur JE Kumar H Youssef M Suresh S. Fecal Lcn-2 level is a sensitive biological indicator for gut dysbiosis and intestinal inflammation in multiple sclerosis. Front Immunol (2022) 13:1015372. doi: 10.3389/fimmu.2022.1015372 36341389 PMC9634083

[53] Borton MA Sabag-Daigle A Wu J Solden LM O’Banion BS Daly RA. Chemical and pathogen-induced inflammation disrupt the murine intestinal microbiome. Microbiome (2017) 5:47. doi: 10.1186/s40168-017-0264-8 28449706 PMC5408407

[54] Chassaing B Srinivasan G Delgado MA Young AN Gewirtz AT Vijay-Kumar M. Fecal lipocalin 2, a sensitive and broadly dynamic non-invasive biomarker for intestinal inflammation. PloS One (2012) 7:e44328. doi: 10.1371/journal.pone.0044328 22957064 PMC3434182

[55] Rima B Collins P Easton A Fouchier R Kurath G Lamb RA. ICTV virus taxonomy profile: pneumoviridae. J Gen Virol (2017) 98:2912–3. doi: 10.1099/jgv.0.000959 PMC5775899 29087278

[56] Schildgen* O Simon A Williams J. Animal models for human Metapneumovirus (HMPV) infections. Vet Res (2007) 38:117–26. doi: 10.1051/vetres:2006051 17181987

[57] Lê VB Dubois J Couture C Cavanagh M-H Uyar O Pizzorno A. Human metapneumovirus activates NOD-like receptor protein 3 inflammasome *via* its small hydrophobic protein which plays a detrimental role during infection in mice. PloS Pathog (2019) 15:e1007689. doi: 10.1371/journal.ppat.1007689 30964929 PMC6474638

[58] González LA Melo-González F Sebastián VP Vallejos OP Noguera LP Suazo ID. Characterization of the anti-inflammatory capacity of IL-10-producing neutrophils in response to streptococcus pneumoniae infection. Front Immunol (2021) 12:638917. doi: 10.3389/fimmu.2021.638917 33995357 PMC8113954

[59] Sencio V MaChado MG Trottein F. The lung-gut axis during viral respiratory infections: the impact of gut dysbiosis on secondary disease outcomes. Mucosal Immunol (2021) 14:296–304. doi: 10.1038/s41385-020-00361-8 33500564 PMC7835650

