# Supplementary figures and images for: Human metapneumovirus respiratory infection affects both innate and adaptive intestinal immunity
Source: Front Immunol. 2024 Feb 2;15:1330209. doi: 10.3389/fimmu.2024.1330209 (PMC10884822; doi:10.3389/fimmu.2024.1330209)

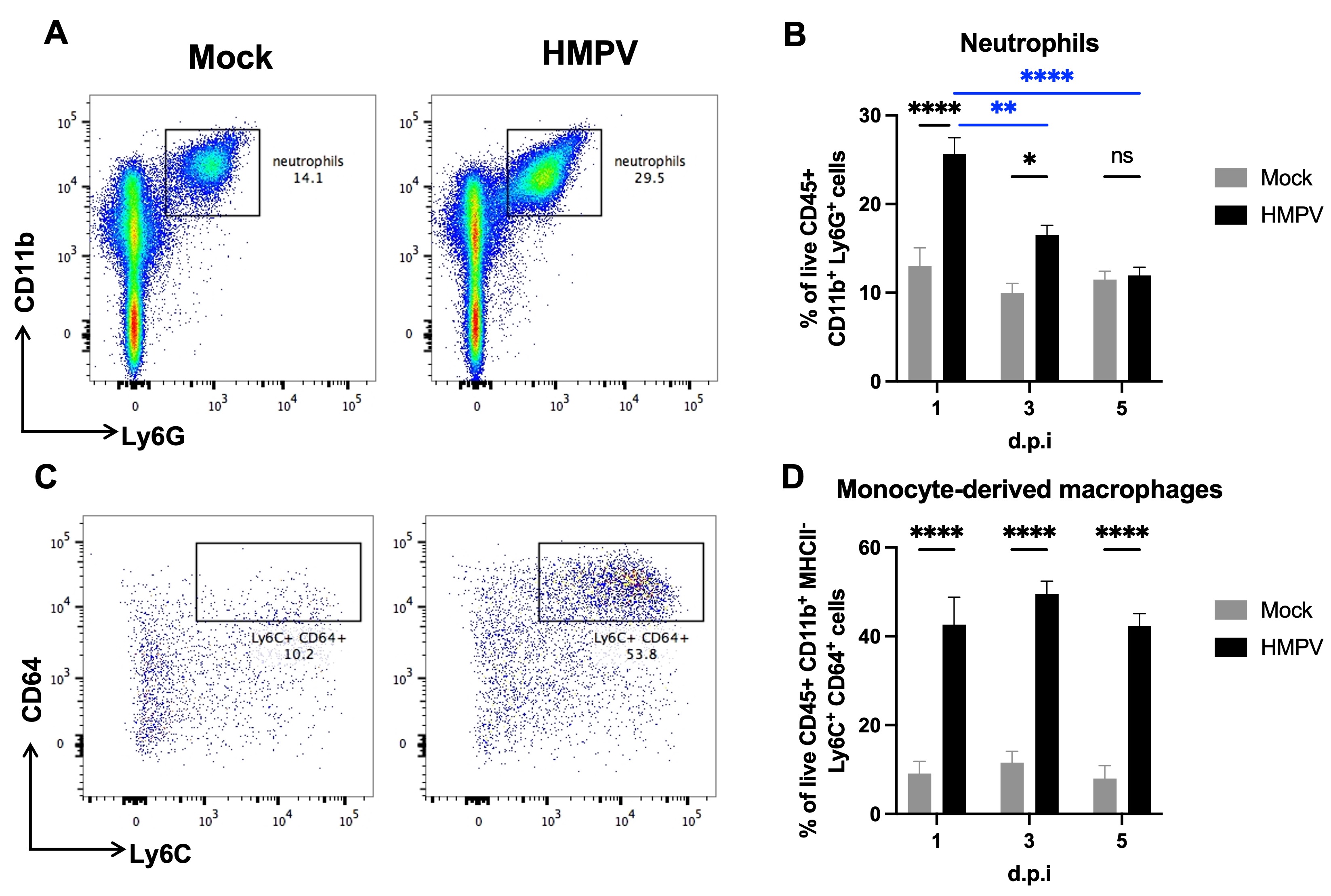

Supplement: Supplementary Figure 1 — HMPV-infected mice exhibit increased recruitment of lung neutrophils and monocyte-derived macrophages. Lung cells were analyzed at different time points following HMPV infection using flow cytometry. A) Representative plots of neutrophils (gated as live CD45+ CD11b+ Ly6G+ cells) in Mock and HMPV-infected mice at day 1 post-infection. B) Frequency of neutrophils. C) Representative plots of monocyte-derived macrophages (gated as live CD45+ CD11b+ SiglecF- MHCII+ Ly6C+ CD64+ cells) in Mock and HMPV-infected mice at day 1 post-infection. D) Frequency of monocyte-derived macrophages. Mock-treated mice are shown in grey, and HMPV-infected mice are shown in black. All data are shown as mean ± SEM and are representative of two independent experiments (mock-treated mice n=5-6 per group; HMPV-infected mice n=6 per group). Statistical differences were evaluated by a two-way ANOVA comparing the means of all the columns and rows corresponding to each group, followed by a post hoc Tukey test (*p<0.05 and **p<0.01). [file Image_1.jpeg]

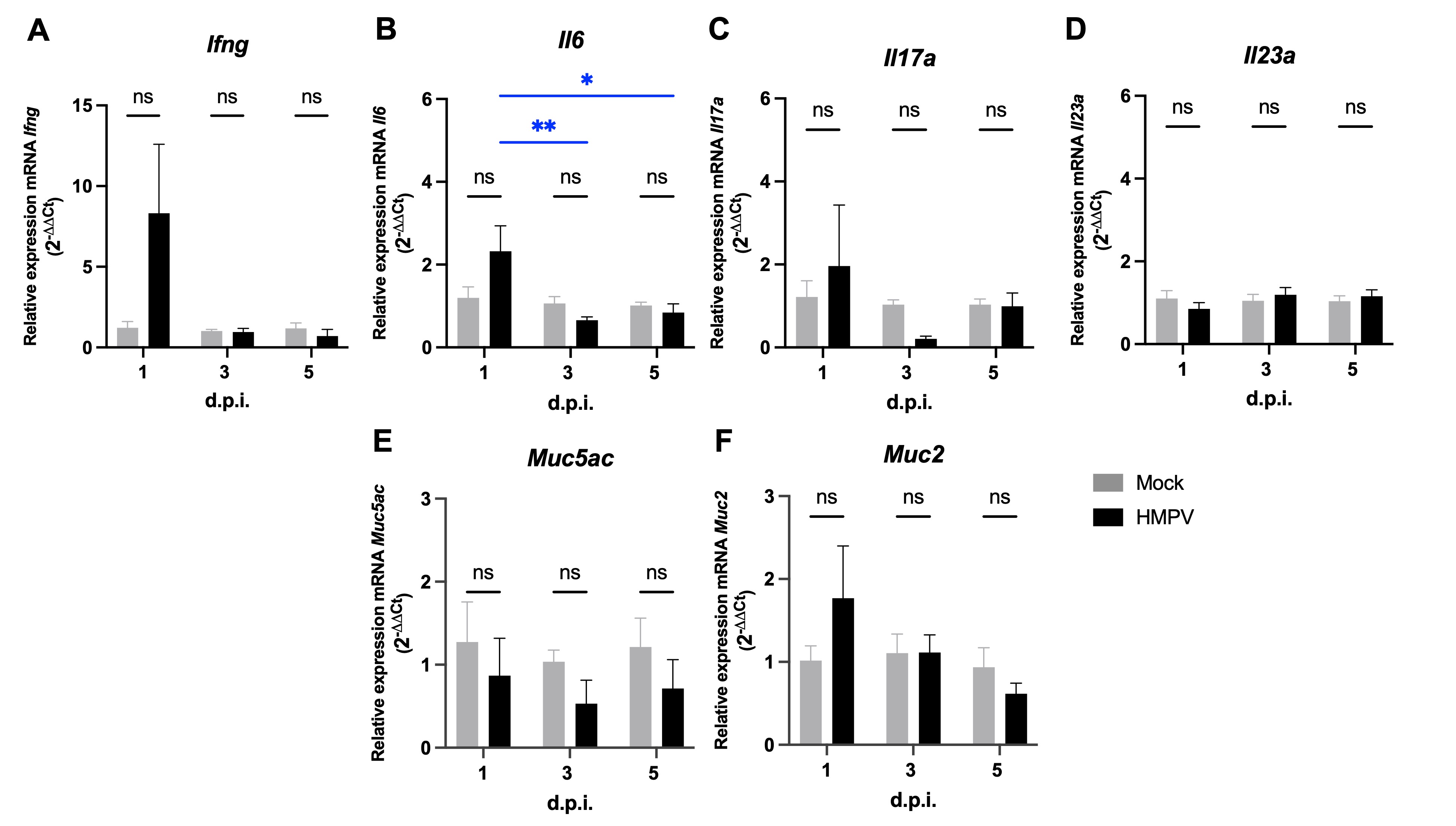

Supplement: Supplementary Figure 2 — HMPV-infected mice do not exhibit changes in pro-inflammatory cytokines in the small intestine. Transcription of different pro-inflammatory cytokines was assessed in the small intestine of mock-treated and HMPV-infected mice at different time points. A) Relative expression of Ifng. B) Relative expression of Il6. C) Relative expression of Il17a. D) Relative expression of Il23a. E) Relative expression of Muc5ac. F) Relative expression of Muc2. Data representative of two independent experiments (mock-treated mice n=5-6 per group; HMPV-infected mice n=6 per group). Mock-treated mice are shown in grey, and HMPV-infected mice are shown in black. All data are shown as mean ± SEM and are representative of two independent experiments (mock-treated mice n=5-6 per group; HMPV-infected mice n=6 per group). Statistical differences were evaluated by a two-way ANOVA comparing the means of all the columns and rows corresponding to each group, followed by a post hoc Tukey test (*p<0.05 and **p<0.01). [file Image_2.jpeg]

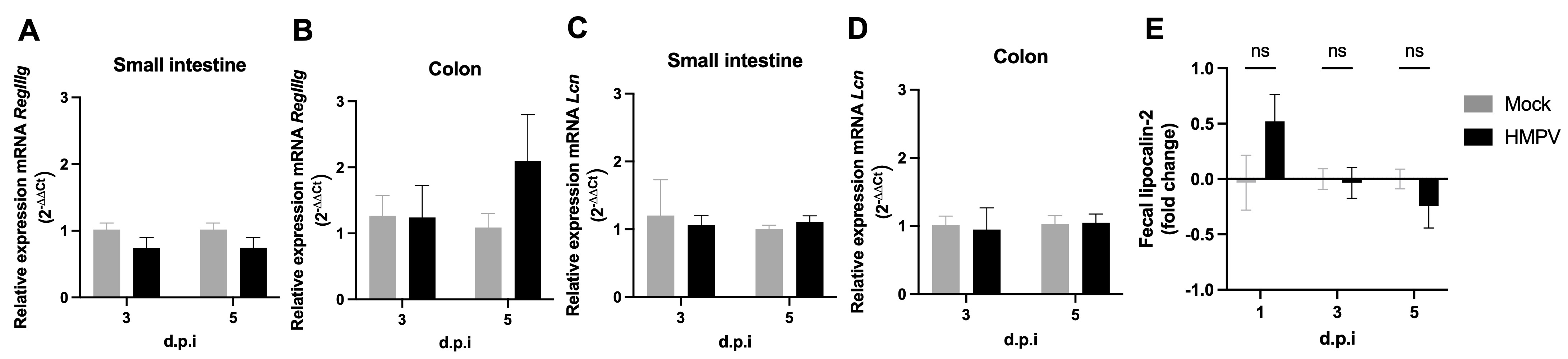

Supplement: Supplementary Figure 3 — HMPV-infected mice do not exhibit changes in the transcription of antimicrobial peptides in the small intestine and colon and fecal levels of Lipocalin-2. Expression at the RNA level of different pro-inflammatory cytokines was assessed in the small intestine and colon of mock-treated and HMPV-infected mice at different time points. A) Relative expression of RegIIIg in the small intestine. B) Relative expression of RegIIIg in the colon. C) Relative expression of Lcn2 in the small intestine. D) Relative expression of Lcn2 in the colon. E) Fold change of fecal lipocalin-2 levels measured by ELISA in fecal supernatants. Mock-treated mice are shown in grey, and HMPV-infected mice are shown in black. All data are shown as mean ± SEM and are representative of two independent experiments (mock-treated mice n=5-6 per group; HMPV-infected mice n=6 per group). Statistical differences were evaluated by a two-way ANOVA comparing the means of all the columns and rows corresponding to each group, followed by a post hoc Tukey test. [file Image_3.jpeg]

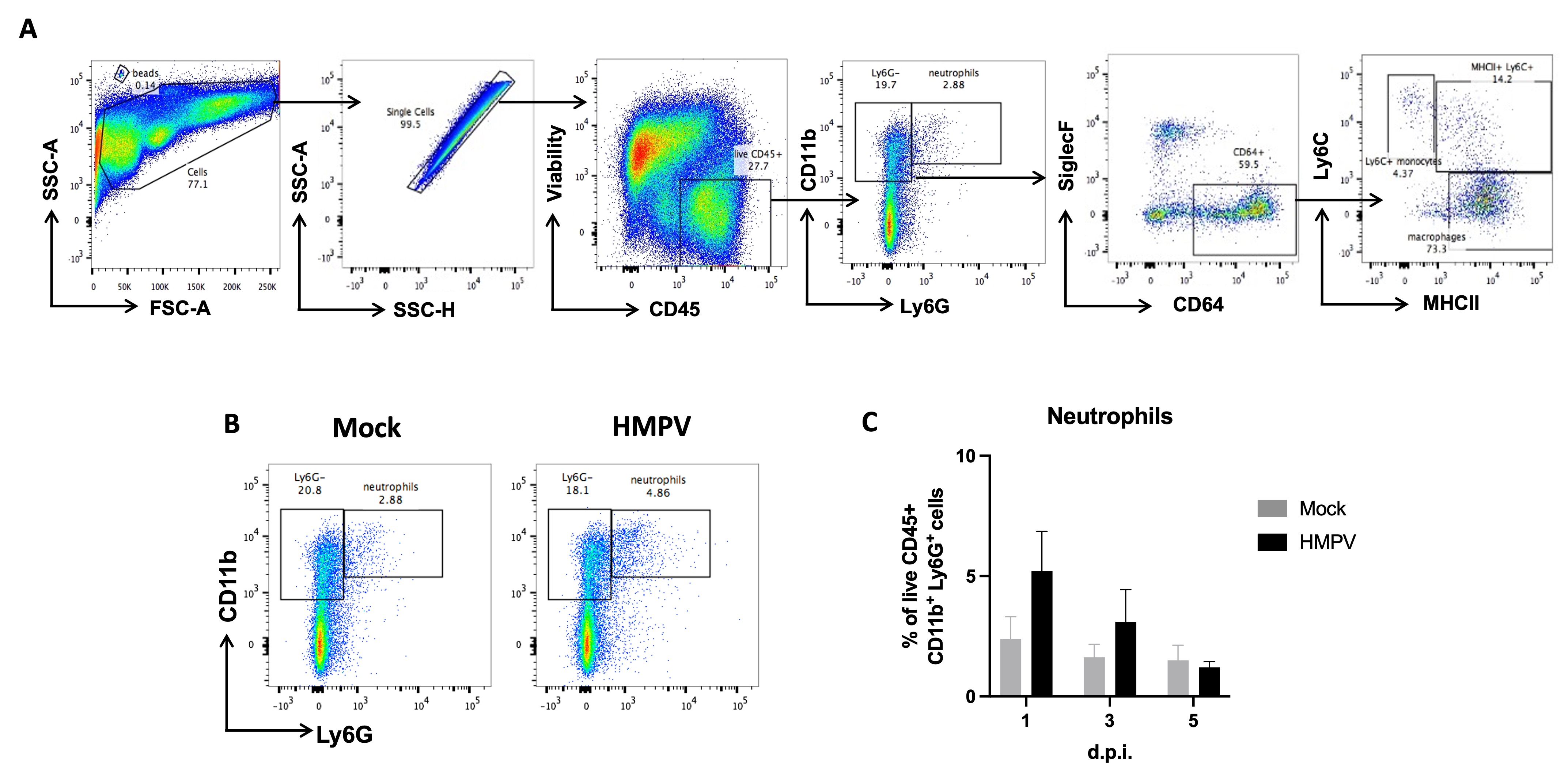

Supplement: Supplementary Figure 4 — HMPV-infected mice do not exhibit increased recruitment of neutrophils into the colon at different post-infection time points. Colon lamina propria cells were analyzed at different time points following HMPV infection using flow cytometry. A) Gating strategy for neutrophils (live CD45+ CD11b+ Ly6G+ cells), macrophages (live CD45+ CD11b+ CD64+ Ly6C- MHCII+ cells) and monocytes (live CD45+ CD11b+ CD64+ Ly6C+ MHCII-/+ cells). B) Representative plots of neutrophils (gated as live CD45+ CD11b+ Ly6G+ cells) in Mock and HMPV-infected mice at day 1 post-infection. C) Frequency of colonic neutrophils. Mock-treated mice are shown in grey, and HMPV-infected mice are shown in black. All data are shown as mean ± SEM and are representative of two independent experiments (mock-treated mice n=5-6 per group; HMPV-infected mice n=6 per group). Statistical differences were evaluated by a two-way ANOVA comparing the means of all the columns and rows corresponding to each group, followed by a post hoc Tukey test. [file Image_4.jpeg]

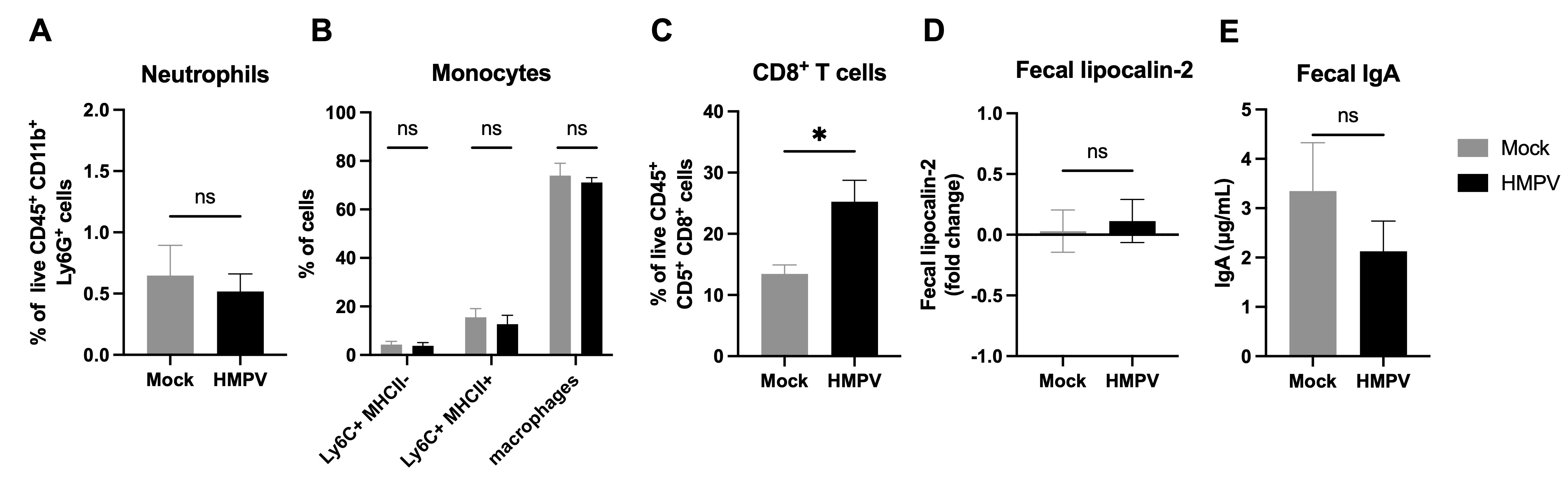

Supplement: Supplementary Figure 5 — HMPV-infected mice do not exhibit changes in the expression of antimicrobial peptides in the small intestine and colon. Colon lamina propria cells were analyzed at different time points following HMPV infection using flow cytometry. A) Frequency of colonic neutrophils. B) Frequency of colonic monocytes and macrophages. C) Frequency of CD8+ T cells. Expression of lipocalin-2 and IgA was measured in fecal supernatants by ELISA. D) Fold change of fecal lipocalin-2 levels. E) Levels of free fecal IgA. Mock-treated mice are shown in grey, and HMPV-infected mice are shown in black. All data are shown as mean ± SEM and are representative of two independent experiments (mock-treated mice n=5 per group; HMPV-infected mice n=6 per group). Statistical differences were evaluated using an unpaired t test. [file Image_5.jpeg]

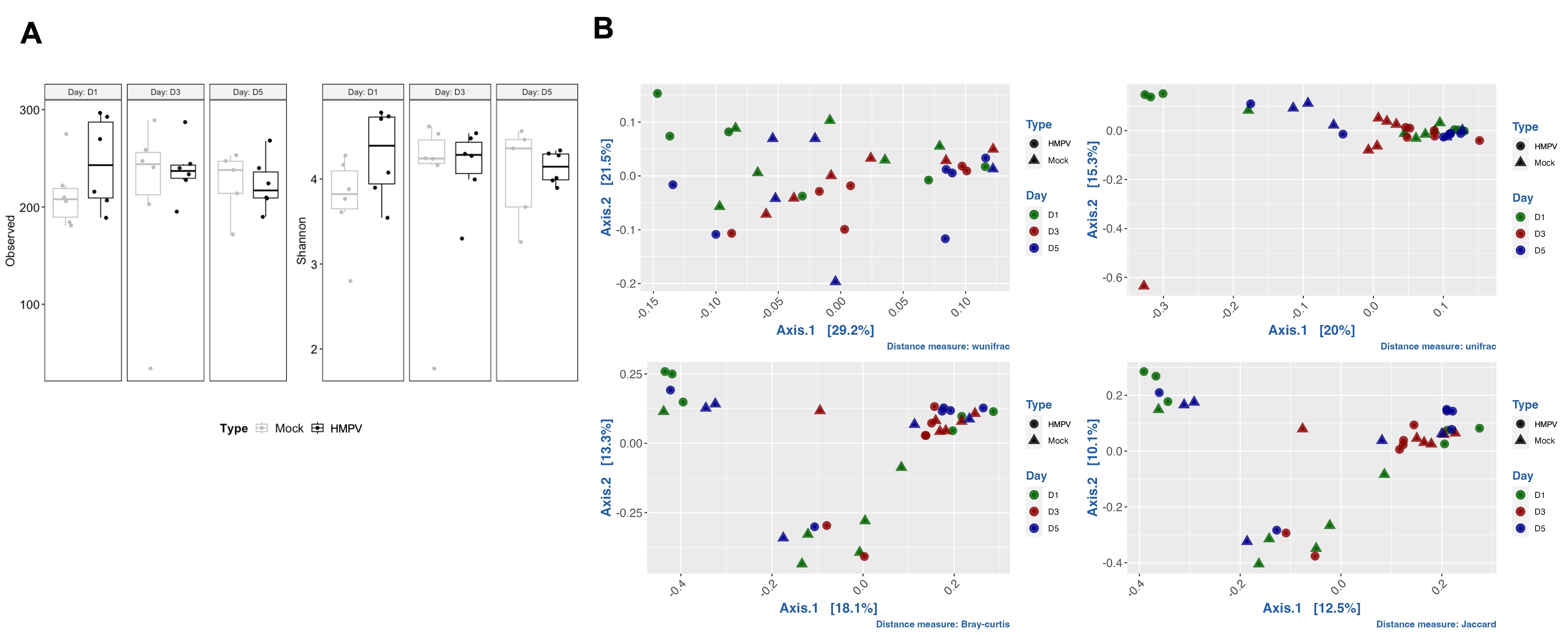

Supplement: Supplementary Figure 6 — HMPV-infected mice do not exhibit fecal microbiota β diversity alterations. A) Alpha diversity of fecal Mock and HMPV samples at different timepoints, analyzed by Observed and Shannon Index. Mock-treated mice are shown in grey, and HMPV-infected mice are shown in black. B) PCoA analysis of all samples by group (mock and HMPV) and timepoint. Mock samples are shown as triangles, and HMPV samples are shown as circles. Time points are shown in green (day 1), red (day 3), and blue (day 5). PERMANOVA test was conducted using distance matrices (HMPV samples between days: Unifrac, Jaccard y Bray-Curtis, HMPV, p < 0.05; Mock samples between days: Unifrac (p < 0.001); wUnifrac (p < 0.05); Bray- Curtis (p < 0.001) and Jaccard (p < 0.001); no significant differences between mock and HMPV samples at any timepoint). Data are representative of two independent experiments (mock-treated mice n=5-6 per group; HMPV-infected mice n=6 per group). [file Image_6.jpeg]
